# Natural Language Processing Applied to Psychiatric Clinical Notes: Scoping Review

**DOI:** 10.2196/91249

**Published:** 2026-07-10

**Authors:** Shuying Rao, Xi'ang Chen, Guifeng Deng, Junyi Xie, Tiecheng Jiang, Tao Li, Yaoyun Zhang, Haiteng Jiang

**Affiliations:** 1 School of Brain Science and Brain Medicine, and Liangzhu Laboratory, Zhejiang University School of Medicine, Affiliated Mental Health Center and Hangzhou Seventh People's Hospital, 305 Tianmushan Road, Xihu District, Hangzhou, Zhejiang, 310058, China, 86 0571-87071107; 2 College of Biomedical Engineering and Instrument Science, Zhejiang University, Hangzhou, Zhejiang, China; 3 MOE Frontier Science Center for Brain Science and Brain-machine Integration, Zhejiang University, State Key Laboratory of Brain-machine Intelligence, Hangzhou, Zhejiang, China; 4 Zhejiang Key Laboratory of Clinical and Basic Research for Psychiatric Diseases, Hangzhou, Zhejiang, China; 5 Department of Biomedical Engineering, Shenyang University of Technology, Shenyang, Liaoning, China; 6 The University of Texas at Dallas, Richardson, TX, United States

**Keywords:** psychiatric clinical notes, electronic health records, natural language processing, large language models, information extraction

## Abstract

**Background:**

Psychiatric clinical notes in electronic health records (EHRs) provide rich longitudinal information that can support clinical decision-making. Using historical medical data can enable earlier identification of mental illness, better characterization of disease trajectories, and more personalized treatment planning. Natural language processing (NLP) transforms these unstructured notes into analyzable representations for research and care.

**Objective:**

This study aims to systematically summarize NLP methodologies for psychiatric clinical notes, compare major modeling paradigms and application areas, and highlight emerging large language model (LLM) trends, key challenges, and future research directions.

**Methods:**

Following the PRISMA-ScR (Preferred Reporting Items for Systematic Reviews and Meta-Analyses Extension for Scoping Reviews) guidelines, a literature search was conducted for articles on NLP methods based on psychiatric clinical notes published from January 2021 to December 2025 in Ovid MEDLINE, Ovid EMBASE, PubMed, Scopus, Web of Science, the ACM Digital Library, and ScienceDirect. This scoping review analyzed NLP methods applied to psychiatric clinical notes, focusing on major trends, identifying suitable features for traditional machine learning (ML)–based models, applications of pretrained language models (PLMs), and key challenges. Approaches were categorized as rule-based, traditional ML, hybrid, deep learning (DL), and LLM-based methods across information extraction and text classification tasks.

**Results:**

In total, 101 studies were eligible for inclusion. Rule-based methods (n=36) and hybrid approaches (n=34) remained the most widely used techniques, largely favored for their interpretability in handling nuanced, subjective clinical notes. These were followed by DL (n=15), traditional ML (n=10), and LLM-based approaches (n=6). Traditional ML studies relied heavily on engineered features, which could be grouped into 5 broad categories: domain knowledge features, lexical and statistical features, vector-based semantic features, emotion-related features, and temporal features. PLMs improved performance mainly through domain adaptation and task-specific fine-tuning, enhancing the handling of psychiatric language, medical terminology, and clinical note structure. LLM-based studies, although still limited in number, indicated a growing shift toward generative and reasoning-based applications.

**Conclusions:**

Hybrid NLP approaches remain dominant, combining domain rules with ML for extraction and classification. DL approaches continue to advance, with domain adaptation supporting medical terminology and clinical semantics. LLMs may further automate complex workflows via zero-shot capabilities and reasoning, alongside growing interest in temporal modeling and multimodal integration. Key future needs include improved generalizability across institutions, privacy protection, and careful attention to ethical implications in clinical deployment.

## Introduction

Mental illness, also known as psychiatric disorders, is a prevalent problem worldwide and continues to be one of the most serious public health issues [1]. There are many different types of mental illness, including depression, suicidal ideation, bipolar disorder, autism spectrum disorder (ASD), anxiety disorders, schizophrenia, etc. All kinds of mental illness negatively affect an individual’s physical health and well-being, and the COVID-19 epidemic has further exacerbated this problem [2]. According to recent statistics, millions of people worldwide experience one or more psychological disorders [1]. Therefore, it is of great significance to study the development trajectory and potential mechanisms of mental illness based on scientific and objective measurement methods, and to promote the development of early diagnosis, personalized medicine, and precision treatment strategies for mental illness.

Language plays a central role in mental health, serving as a medium for expressing symptoms, delivering therapy, and assessing clinical conditions. Traditionally, language analysis in psychiatry has relied on expert opinions, clinical ratings, and manual methods, which are often subjective, incomplete, or prone to inaccuracies. Automated language analysis offers a transformative opportunity to shift from subjective clinical judgment to “measurement-based care” [3], enabling robust, quantitative, and scalable tracking of language variables. This advancement has the potential to revolutionize psychiatric practice and research.

Electronic health records (EHRs) are a rich source of health care data and have been widely used to record patient medical histories [4]. Psychiatric EHRs often include narrative clinical notes that contain valuable information for advancing clinical research and health care [5]. However, much of this clinical information remains locked in unstructured text [6], posing challenges for systematic analysis. In the field of psychiatry, standardized measures of patients are used inconsistently and infrequently in clinical practice. Due to the heterogeneity and complexity of clinical notes, along with its diverse application scopes, the application of natural language processing (NLP) in clinical notes still needs to be further explored.

The rise of statistical NLP in the 1990s [7] and recent advances in deep learning (DL) technology [8,9] have influenced the methods used in clinical notes analysis. A pivotal turning point in this trajectory was the introduction of the Transformer architecture, which enabled a new modeling paradigm: pretrained language models (PLMs), such as Bidirectional Encoder Representations from Transformers (BERT) [10]. PLMs are first trained on large, general-purpose text corpora via self-supervised objectives, then adapted to specific downstream tasks through fine-tuning — substantially reducing dependence on large manually annotated datasets. With the rapid expansion of computational resources, PLMs were subsequently scaled to billions of parameters, giving rise to large language models (LLMs), such as GPT and Gemini, which demonstrate superior performance across a wide range of NLP tasks, including text classification, entity recognition, summarization, sentiment analysis, and text generation [11]. Clinical applications for clinical notes, such as summarization and information extraction [12,13], have been widely used to assist in disease diagnosis, including suicide screening [14], depression identification [15], and mental state prediction [16].

Several recent reviews have explored various aspects of NLP in mental health, each with distinct focuses. Some reviews are highly specialized, concentrating on specific aspects such as mental illness detection [17] or intervention tools [18]. Other reviews do not incorporate the recent breakthroughs, such as the development and application of attention mechanisms, transformers, and pretrained LLMs. Reviews such as Le Glaz et al [19] primarily focus on traditional NLP and machine learning (ML) techniques applied across mental health domains. Meanwhile, Jin et al [20] provide a high-level overview of LLM applications and performance metrics without diving into the specific methodological challenges of working with clinical notes. Given these gaps, this scoping review aims to provide a comprehensive and methodologically detailed examination of NLP methods applied specifically to psychiatric clinical notes. We focus on the latest trends, tools, and challenges in using NLP for mental illness research based on clinical notes.

This scoping review aimed to address the following research questions:

1. What are the major NLP trends and methods for clinical notes analysis in psychiatric disorders?
2. In traditional ML-based models, which features of clinical notes are suitable for extraction for downstream research on mental illness?
3. How to apply PLMs to improve the performance of text-based models in the field of mental illness?
4. What are the main challenges and future directions for NLP in clinical notes of psychiatric disorders?

## Methods

### Design and Protocol Registration

This scoping review was conducted in accordance with the Joanna Briggs Institute guidance for scoping reviews and reported in compliance with the PRISMA-ScR (Preferred Reporting Items for Systematic Reviews and Meta-Analyses extension for Scoping Reviews) [21-23]. A formal protocol was not prospectively registered. The review protocol was retrospectively registered in the Open Science Framework [24]. The completed PRISMA-ScR checklist can be found in Checklist 1.

Our search included any document published from January 2021 to December 2025. We selected 2021 as the starting year to position this review as an update to prior broad mental health NLP reviews and to focus on the most recent methodological phase of the field.

### Information Sources and Search Strategy

A systematic electronic search was developed with an experienced librarian and implemented across 7 databases: Ovid MEDLINE, Ovid EMBASE, PubMed, Scopus, Web of Science, the ACM Digital Library, and ScienceDirect. The search was first performed on January 16, 2024, and then updated with a second search on December 15, 2025.

The search strategy combined controlled vocabulary terms, where available, and free-text terms covering 3 core concepts: (1) psychiatric or mental disorders, (2) clinical notes or electronic health records, and (3) NLP or text mining. Database-specific syntax, subject headings, field tags, and adjacency operators were adapted for each source while preserving the same conceptual structure across databases. The full search strategies for all databases are reported in Multimedia Appendix 1.

All retrieved citations were exported to Zotero. Duplicate records were removed in 2 stages: (1) automated deduplication using bibliographic fields such as title, author, year, journal, and DOI or PMID, followed by (2) manual review of potential duplicate pairs.

### Inclusion and Exclusion Criteria

We included English-language full-text journal articles and full conference or workshop papers that reported completed studies using NLP methods applied to English psychiatric clinical notes, and that made a methodological contribution. Studies using quantitative, qualitative, or mixed-method designs were eligible if they described completed research.

We excluded abstract-only publications, study protocols, review articles, editorials, and papers without sufficient full-text methodological detail. We also excluded studies in which the primary text source was not psychiatric clinical notes; examples included interviews, speech or voice data, social media posts, or general clinical text unrelated to mental health. Studies were also excluded if they did not apply NLP methods or if their application was not specific to psychiatry or mental illness (eg, deidentification or redundancy removal).

### Study Selection and Screening Process

Initial screening of the titles and abstracts was conducted independently by 2 reviewers either (SR and TJ) or (GD and JX). The records were divided between 2 reviewer pairs (SR with TJ, and GD with JX), and each record was assessed by 1 reviewer pair. At this stage, each article was categorized into one of the following groups: (1) fully met the inclusion criteria, (2) did not analyze clinical data, (3) did not use NLP methods, (4) did not focus on mental illness, and (5) had unclear eligibility for inclusion. To maximize sensitivity, records were advanced to full-text review whenever eligibility could not be determined from the title and abstract alone.

Full-text eligibility assessment followed the same procedure, with each report independently assessed by 2 reviewers within 1 reviewer pair. At this stage, all inclusion criteria had to be met for a study to be retained in the review. Disagreements at either stage were first resolved through discussion within the reviewer pair; if consensus could not be reached, a third reviewer (XC) adjudicated the final decision.

### Data Collection

The final data collection form used for peer-reviewed articles is shown in Table S1 in Multimedia Appendix 2. The information for each study included data sources, sample information, regions, NLP tasks, applied NLP methods, application domain, and psychiatric categories. These articles were divided into 5 parts (each part included 20 or 21 papers). A total of 5 researchers (SR, XC, GD, JX, and TJ) independently extracted data from each part of the papers. To ensure the accuracy of data extraction, we conducted a double-check process. The researchers cross-checked each other’s work, reviewing the data extraction process.

For this scoping review, we developed a categorization framework based on NLP methodology approach applied for psychiatric clinical notes: rule-based, traditional ML, hybrid, DL, and LLM-based methods. Definitions of the key terms used throughout this review are provided in Multimedia Appendix 3.

## Results

### Overview

**Figure 1.**
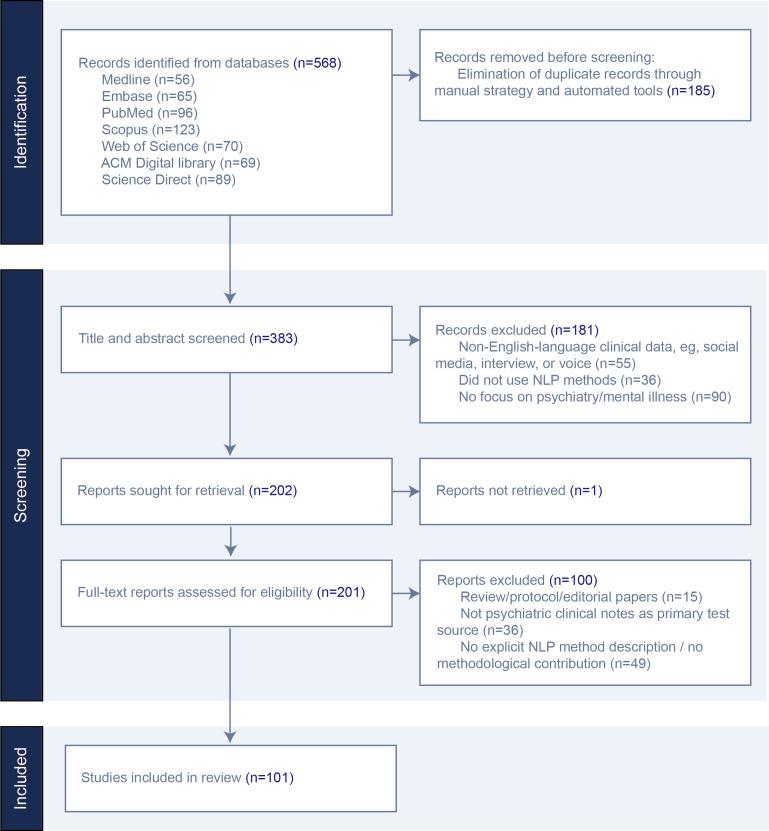
PRISMA (Preferred Reporting Items for Systematic Reviews and Meta-Analyses) flowchart of search history. EHR: electronic health records; NLP: natural language processing.

The initial search yielded 383 records after deduplication. Following title and abstract screening, 181 records were excluded, leaving 202 reports for full-text retrieval. Of these, 1 report could not be retrieved, and the remaining 201 were assessed for eligibility. Full-text screening resulted in the exclusion of a further 100 articles, with reasons detailed in Figure 1. Ultimately, 101 studies [25-125] were included in the scoping review, as shown in Multimedia Appendix 4. The results of the search and the study inclusion process are presented in the PRISMA (Preferred Reporting Items for Systematic Reviews and Meta-Analyses) flowchart in Figure 1 [126].

**Figure 2.**
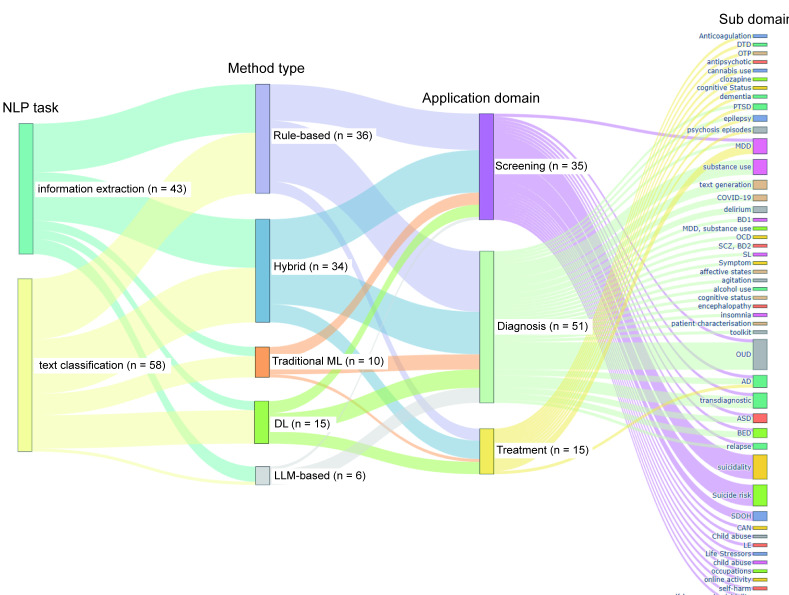
Sankey diagram of natural language processing (NLP) tasks, methods, and clinical applications across the 101 included studies. The diagram is read from left to right, linking NLP task categories (information extraction and text classification) to method types (rule-based, hybrid, traditional machine learning (ML), deep learning (DL), and large language model [LLM]–based approaches), then to the 3 main clinical application domains (screening, diagnosis, and treatment), and finally to specific psychiatric subdomains. The width of each flow is proportional to the number of studies following that pathway, thereby showing how specific NLP tasks and methods connect to downstream clinical applications. AD: Alzheimer disease; ASD: autism spectrum disorder; BD1: behavioral disturbance; BD2: bipolar disorders; BED: binge-eating disorder; CAN: child abuse and neglect; CVD: cardiovascular disease; DTD: difficult-to-treat depression; LE: life events; MDD: major depressive disorder; OCD: obsessive-compulsive disorder; OTP: opioid treatment program; OUD: opioid use disorder; PTSD: posttraumatic stress disorder; SCZ: schizophrenia; SDOH: social determinants of health; SL: stigmatizing language.

Across the 101 included studies [25-125], the identified tasks were broadly grouped into information extraction (IE) and text classification (TC), implemented using rule-based, traditional ML, hybrid, DL, and LLM-based approaches. Figure 2 presents a Sankey diagram [127] illustrating the relationships among NLP tasks, methods, and application domains in mental health. Specifically, the included studies were organized into three major application domains: screening, diagnosis, and treatment. Screening-related studies mainly focused on suicidal ideation, suicide risk, crisis prediction, and related risk detection; diagnostic applications involved symptom identification, disease classification, phenotype characterization, and comorbidity recognition; and treatment-related studies addressed drug information extraction, treatment quality assessment, and outcome prediction. Together, these pathways highlight the interdisciplinary nature of applying NLP to psychological assessment and intervention by demonstrating how various computational techniques interconnect across different domains.

The following subsections detail the resources (including data sources and knowledge bases) and a systematic review of modeling approaches ranging from rule-based and traditional ML to hybrid, DL, and the latest LLM-based approaches. Finally, we address model evaluation metrics and performance. Although many techniques overlap with general clinical NLP, the distinctive characteristics of psychiatric notes and mental health care challenges require specialized resources, methods, and considerations.

### Resources

Clinical notes in the reviewed studies were primarily sourced from medical institutions, with access typically requiring data use applications and institutional review board approval due to privacy concerns. Among the 101 included studies, data were drawn from 42 medical institutions and only one public database, the MIMIC-III dataset [128], underscoring the limited availability of openly accessible psychiatric EHR resources. Specialized psychiatric data sources, particularly the South London and Maudsley NHS Foundation Trust [129] and the US Department of Veterans Affairs (VA) [130], were among the most frequently used, appearing in 24 and 9 studies, respectively. In addition, Rush University Medical Center’s Substance Use Intervention Team (SUIT) [131] program contributed data to five studies on opioid addiction. Overall, the heavy reliance on institution-specific proprietary databases indicates substantial fragmentation in the current resource landscape, which may hinder reproducibility, external validation, and cross-institutional collaboration. A detailed distribution of data sources is provided in Multimedia Appendix 5.

The Observational Medical Outcomes Partnership (OMOP) common data model is an open standard designed to standardize observational data, enabling efficient and reliable analyses [132]. In [25,26], the OMOP common data model was used to harmonize clinical data, including diagnostic codes, demographics, and clinical notes. Similarly, the Health Level 7 (HL7) Fast Healthcare Interoperability Resources (FHIR) standard facilitates health care data exchange using modern web technologies, organizing data into modular “resources” accessible via RESTful APIs [133]. Afshar et al [27] used HL7 data standards to transfer EHR data to the cloud, supporting the deployment of a real-time NLP CDS tool for opioid misuse screening. However, the adoption of these standards in mental health remains limited due to challenges such as customization requirements, integration complexity, and privacy concerns. Future advancements in data-sharing frameworks and public dataset availability may enhance research in this field.

Standard terminology sets are foundational for NLP in psychiatric clinical notes by providing unified concepts and formats that support interoperability across institutions and systems. Commonly used terminologies include the Unified Medical Language System (UMLS) [134], Systematized Nomenclature of Medicine–Clinical Terms (SNOMED-CT) [135], *International Classification of Diseases, Ninth Revision* (*ICD-9*) and *International Classification of Diseases, Tenth Revision* (*ICD-10*) [136], and RxNorm [137] for drug information. Psychiatric clinical notes analysis also faces distinct needs—complex diagnostic criteria, behavioral assessments, subjective language, strict confidentiality, and longitudinal monitoring—so domain-specific resources are often essential. The *Diagnostic and Statistical Manual of Mental Disorders, Fifth Edition* (*DSM-V*) provides standardized diagnostic criteria for mental disorders [138], while the Addiction Behaviors Checklist (ABC) supports structured assessment of addictive behaviors [139]. Links to these terminology sets are provided in Table 1.

**Table 1.** An overview of the dictionaries and knowledge bases used for extracting information (data) from clinical notes.

Dictionary or knowledge base	Description	Examples
UMLS^a^ [134]	Biomedical thesaurus organized by concept and it links similar names for the same concept	[28]
SNOMED-CT^b^ [135]	A globally accepted medical terminology system	[29]
*ICD^c^* [136]	Knowledge on the extent, causes, and consequences of human disease and death worldwide	[30]
*DSM*^d^ [138]	Standardized classification and criteria for diagnosing mental disorders	[31]
RxNorm [137]	A standardized nomenclature for clinical drugs	[32]
ABC^e^ [139]	A scale commonly used to assess an individual’s addictive behavior	[33]

a UMLS: Unified Medical Language System.

b SNOMED-CT: Systematized Nomenclature of Medicine–Clinical Terms.

c *ICD*: *International Classification of Diseases.*

d *DSM*: *Diagnostic and Statistical Manual of Mental Disorders*.

e ABC: Addiction Behaviors Checklist.

### Rule-Based Approaches

Rule-based approaches use predefined rules and keyword-based features to identify patterns in text [140], offering transparency, traceability, and cost-effectiveness by leveraging domain-specific knowledge without requiring large annotated datasets [141]. These methods often rely on clinical standards, guidelines, curated dictionaries, and knowledge bases, which can be easily updated and adapted [142]. Initial keyword lists are typically derived from expert domain knowledge or standard terminologies like UMLS, SNOMED-CT, and *ICD-9* and *ICD*-*10*, enabling comprehensive coverage of medical concepts. Rule-based NLP pipelines map free-text clinical notes to standardized ontologies (eg, UMLS) through medical concept normalization, identifying terms related to drugs, diagnoses, symptoms, and clinical measurements while expanding searches to related concept groups.

Clinical notes often contain nonstandard language, abbreviations, misspellings, and diverse expressions for the same symptom. Rule-based approaches refine terminology dictionaries using regular expression (RegEx) patterns and iterative feedback from domain experts [34,35]. They also identify modifiers—such as emotions (negation, affirmation), descriptive attributes (severity, duration), and annotation sections (medical history, assessment)—to contextualize concepts and exclude irrelevant mentions [32,36]. Negation detection, often using the NegEx algorithm [143], is a key application. Rule-based methods can also track symptom trajectories, as demonstrated by Young et al [37], who classified and monitored dynamic behavioral phenotypes in ICU patients over time.

However, relying solely on dictionary-based concept recognition cannot capture all essential information in clinical texts. To capture comprehensive information, custom rule sets are often developed, as detailed in [144]. Several NLP systems facilitate rule-based development, including MedLEE [145], MetaMap [146], clinical text analysis and knowledge extraction system (cTAKES) [147], Clinical Language Annotation, Modeling, and Processing Toolkit (CLAMP) [148], and Biomedical Information Collection and Understanding System (BioMedICUS) [149], which are widely used for named entity recognition (NER) and IE in clinical and biomedical research [150].

### Traditional Machine Learning Approaches

Traditional ML refers to a family of statistical and mathematical algorithms that learn patterns from data to make predictions or decisions, but typically require human-guided feature engineering rather than automatically extracting hierarchical representations through multilayered neural networks. In TC tasks, traditional ML models such as Conditional Random Fields, support vector machines, structured support vector machines, logistic regression, Bayesian models, and random forests are commonly used. These models learn patterns from input data and labeled outputs without explicit programming [151], with their development process involving data preprocessing, feature extraction, modeling, optimization, and evaluation. Unlike DL, traditional ML requires significant human intervention for feature engineering. As summarized in Table 2, the most suitable features fall into five broad categories: domain knowledge features, lexical and statistical features, vector-based semantic features, emotion-related features, and temporal features. These features capture different aspects of psychiatric clinical notes that are relevant to downstream research. Domain knowledge and lexicon-based features are useful for identifying explicit symptom descriptions and clinically salient terminology; semantic vector representations help normalize variation in narrative expression; emotion-related features, extracted using tools like ABSApp [152], are particularly relevant for affective states and suicide risk [38]; and temporal features are important for modeling symptom progression [39], instability, and longitudinal risk. Together, these findings suggest that traditional ML is especially suited to settings where clinically interpretable features can be engineered from notes to support tasks such as phenotyping, subgroup discovery, outcome prediction, and risk stratification.

**Table 2.** An overview of features used in traditional machine learning-based models.

Features	Description	Examples
Domain knowledge features		
UMLS^a^	UMLS is a set of key terminology, coding standards, and associated resources related to biomedical information.	[28,29]
Lexical and statistical features		
BoW^b^	The simplest form of text representation using numbers of vocabularies.	[40]
n-gram	N-gram is a contiguous sequence of n words.	[41]
TF-IDF^c^	TF-IDF reflects the importance of the word in the document.	[31,42]
Vector-based semantic features		
Word embedding	The vector-based representation of words. Examples: word2vec, GloVe.	[43]
Emotion-related features		
Sentiment scores	Determining the sentiment polarity of texts (positive, negative, or neutral)	[38,44]
Temporal features		
Time features	Focusing on the time-related features, like time interval.	[39]

a UMLS: unified medical language system

b BoW: Bag of Words

c TF-IDF: term frequency–inverse document frequency

Most ML research in this field relies on supervised learning, which requires high-quality labeled data for effective model training. However, data labeling is time-consuming and challenging. Unsupervised learning methods, such as clustering [153] and latent Dirichlet allocation (LDA) topic modeling [45], can extract useful patterns without labeled data and may complement supervised classifiers [46]. Clustering methods, such as non-negative matrix factorization (NMF), can differentiate patient subgroups based on text features. Zhao et al [31] used TF-IDF to normalize ASD terms in clinical texts, revealing distinct ASD subgroups through clustering. Topic modeling, an unsupervised technique for identifying thematic patterns in text [154], represents topics as probability distributions over words. LDA topic modeling has successfully identified insomnia factors within military health systems [45] and transformed unstructured “chief complaints” into quantitative symptom clusters (eg, somatic or cognitive distress) [47]. Additionally, dynamic topic modeling (LDASeq) models how word distributions evolve over time by inducing conditional dependence between sequential intervals. By tracing the longitudinal prominence of themes such as “Suicide” or “Medication,” Levis et al [48] provide a mechanism for monitoring clinical “lability” and time-sensitive risk fluctuations in high-risk populations, offering insights into the dynamic nature of suicide risk that traditional static variables cannot capture.

### Hybrid Approaches

The hybrid approach integrates rule-based and ML methods within a single system. In terminal hybrid models, rule-based systems perform feature extraction, producing structured outputs that serve as inputs for ML models. Rule-based methods, leveraging dictionary lookup and pattern recognition, facilitate medical concept normalization. Feature engineering then vectorizes extracted data for ML applications. Ontologies further enhance NLP by capturing related concepts and contextual nuances, such as negation and speculation in clinical notes. For instance, phrases like “patient denies memory loss” or “possible onset of dementia” are accurately processed. IY Oh et al [49] applied this approach to extract clinical phenotypes from unstructured data, which was then used to train ML models for predicting Alzheimer disease progression and identifying modifiable risk factors.

Hybrid approaches also enhance ML-driven lexicon expansion and disambiguation. Developed open-source NLP software such as NimbleMiner [155] allows users to mine clinical texts to quickly discover large synonym vocabularies containing abbreviations and spelling errors based on word embedding models. For concept normalization, the Medical Concept Annotation Toolkit (MedCAT) [156] uses self-supervised embeddings to disambiguate candidates detected via dictionaries, accurately mapping mentions to the SNOMED-CT ontology even amidst linguistic noise.

Foundational infrastructures like the General Architecture for Text Engineering [157] facilitate hybrid NLP by enabling the integration of rule-based transducers, such as Java Annotation Patterns Engine, with diverse ML resources. A key application is TextHunter [158], which powers the CRIS-CODE project [159] by combining SVM for sentence classification with the rule-based ConText algorithm. By automating the detection of negation, temporality, and subject, TextHunter has successfully extracted over 40 mental health symptoms with a 90% median precision, supporting transdiagnostic research [50,51] and dynamic psychosis risk prediction [52]. Moreover, the CLARK [160] toolkit democratizes these hybrid methods for non-experts by allowing users to define clinical features through RegEx, which are then transformed into feature vectors for downstream ML classifiers. CLARK has demonstrated robust performance in identifying depression and substance use disorder diagnoses within medically complex populations [53]. Lately, VIEWER [54] enhanced mental health care by integrating hybrid NLP into visual analytics, providing a longitudinal, data-driven perspective of patient journeys. An overview of several prominent clinical NLP toolkits in psychiatry based on these hybrid approaches is provided in Table 3.

**Table 3.** An overview of clinical natural language processing toolkits in psychiatry based on hybrid approaches.

Toolkit	Description	Rule-based method	ML^a^ method	Examples
GATE^b^ [157]	A modular infrastructure for the full lifecycle of text analytics	JAPE^c^ grammars for pattern matching and gazetteers	Pluggable models (SVM^d^, Weka, DL)^e^	[55]
TextHunter [158]	A suite designed for psychiatric concept extraction and model building	ConText algorithm for negation and subject detection	SVM for high-precision sentence classification	[51,52]
CLARK [160]	A graphical interface for computable phenotyping by clinical researchers	User-defined RegEx for feature selection	Standard ML classifiers (RF^f^, SVM, Naïve Bayes)	[53]
NimbleMiner [155]	A system for rapid lexicon discovery and mining through word similarity	High-precision regex search using enriched lexicons	Skip-gram word embeddings for finding semantic synonyms	[56]
MedCAT [156]	A toolkit for automated concept linking and disambiguation in clinical notes	Dictionary-based concept candidate detection	Self-supervised embeddings (Word2Vec) for disambiguation	[57]
VIEWER [54]	An interactive visual analytics tool for point-of-care decision support	Pattern matching for symptom and intervention detection	Distributed pipelines for automated clinical data extraction	Not available.

a ML: machine learning.

b GATE: General Architecture for Text Engineering.

c JAPE: Java Annotation Patterns Engine.

d SVM: support vector machine.

e DL: deep learning.

f RF: random forest.

### Deep Learning Approaches

Traditional ML models rely on feature engineering, whereas DL frameworks automatically capture meaningful features without manual intervention [161]. DL, a subfield of ML, learns hierarchical representations through neural networks such as recurrent neural networks (RNNs), convolutional neural networks (CNNs), and transformers.

DL frameworks typically consist of an embedding layer and a classification layer [162]. Embedding techniques range from word embeddings to contextual encoders (eg, BERT [10] and ALBERT [163]). In classification layers, CNNs use convolutional filters to capture spatial relationships and pooling layers to reduce computational complexity. In [58], a CNN-based multilabel classifier was developed to predict alcohol abuse, opioid abuse, and non-opioid substance abuse. This model demonstrated higher sensitivity than single-label classifiers, suggesting that integrating multiple substance abuse screenings into a single model can enhance clinical decision support and reduce alarm fatigue.

Beyond custom architectures, researchers have leveraged modular NLP frameworks to address specific psychiatric tasks with high data efficiency. By fine-tuning ScispaCy [164] to extract “health status” keywords from outpatient notes, Verter et al [59] achieved high precision (>92%) using minimal manual annotations. In addition, Med7 was a DL model originally trained on general physical health data [128], and in [165], it was further fine-tuned on the UK-CRIS database to extract granular pharmacological details (including dosages and titration schedules), thus enabling the comprehensive characterization of treatment resistance in a cohort of over 28,000 patients.

Recent advances in NLP have introduced PLMs — a class of deep neural models pretrained on large text corpora and adapted to specific tasks through fine-tuning. These PLMs, predominantly built on the Transformer architecture, demonstrate strong generalization across tasks and domains [10]. For instance, Ford et al [60] fine-tuned the BERT model for NER to identify 5 key concepts (diagnosis, medication, dosage, signs or symptoms, and substance use), coupled with a contextual classification model to determine the “Status” (eg, has, had, and does not have) and “Experiencer” (eg, patient vs. family member) of the extracted entities. This methodology was integrated into the Akrivia Health database [61] framework to process millions of patient records. Domain-specific adaptations such as BioBERT [166], MentalBERT [167], and Bio_ClinicalBERT [168] have also proven highly effective in clinical settings [62,169]. Building on these discriminative strengths, Xie et al [63] further integrated the generative capabilities of the text-to-text transfer transformer (T5) model [170] to capture complex epilepsy outcomes. T5 was used to transform unstructured notes into precise, structured data regarding seizure frequency and dates, effectively revealing the remitting-relapsing dynamics of epilepsy across large-scale EHRs.

### LLM-Based Approaches

While PLMs transformed the field by facilitating the fine-tuning of broad representations for targeted clinical tasks, the emergence of generative LLMs marks a distinct evolutionary leap [171]. In this review, LLMs refer specifically to PLMs scaled to billions of parameters and built predominantly on decoder-only Transformer architectures. This massive scaling unlocks “emergent” abilities, most notably zero-shot chain-of-thought (CoT) reasoning [172] and in-context learning [173], allowing them to simulate complex clinical decision-making and generate coherent patient narratives without weight updates or extensive task-specific training data [174]. For example, Leng et al [64] demonstrated that GPT-4o, using a “summary of summaries” hierarchical approach, could identify stages of cognitive impairment with an expert concordance rate (Kappa=0.95) that far surpassed BERT-based benchmarks. Moreover, iterative prompt refinement has enabled models like GPT-4 to produce discharge summaries that blinded psychiatry specialists rated as superior to those written by residents [65]. This reasoning capability also facilitates the generation of high-fidelity synthetic data, a crucial development for fields constrained by stringent privacy regulations. Warner et al [66] leveraged 2 local instances of Llama 3.3 (70B) to act as “Interviewer” and “Patient” through CoT-driven interaction; they generated complex synthetic patient profiles that provide a viable alternative to sensitive psychiatric clinical notes for model training.

To bridge the gap between general reasoning and domain-specific precision, researchers have increasingly focused on fine-tuning. Techniques such as low-rank adaptation (LoRA) [175] allow models to master the nuanced lexicon of psychiatry while operating within computational and privacy constraints. Notably, Shukla et al [67] illustrated that smaller, open-source models (eg, Llama-3-8B) fine-tuned via LoRA can outperform massive proprietary models like GPT-4o in specialized tasks, including note proofreading and substance use identification. This trend toward localized optimization is further exemplified by the work of Krishnamoorthy et al [68], who used a fine-tuned Llama 3.3 (70B) to translate complex discharge summaries into patient-friendly language, thereby enhancing health literacy and engagement.

To situate these recent developments within the broader language-model landscape of psychiatric NLP, Table 4 provides a comparative overview of the major language model families applied to psychiatric clinical notes, including both earlier encoder-based PLMs and recent generative LLMs. It demonstrates how foundational open-source encoders are typically fine-tuned for granular extraction, whereas modern scalable models leverage advanced prompting and parameter-efficient tuning to tackle complex reasoning, summarization, and synthetic generation. It also suggests a degree of functional differentiation across model types: general-purpose proprietary LLMs showed their clearest advantages in longitudinal summarization, reasoning-oriented staging, and patient profile generation, whereas domain-adapted or locally fine-tuned open models were often more competitive for tightly bounded extraction, standardization, and institution-specific workflows.

**Table 4.** Overview of language model families applied to psychiatric clinical notes.

Studies	Model family	Representative models	Adaptation strategy
Open-source			
[5,10,60,69]	BERT series	BERT^a^ ClinicalBERT BlueBERT, BioClinicalBERT MentalBERT DementiaBERT	Continued pretraining on clinical or psychiatric corpora Supervised fine-tuning (eg, for Named Entity Recognition) Embedding regularization (eg, triplet-loss)
[7,11,66,68]	LLaMa series	Llama 3.1 Llama 3.2 Llama 3.3	Parameter-efficient tuning (eg, low-rank adaptation) Local instruction tuning (with institution-specific note corpora), Locally deployed multiagent prompting
[70]	FLAN-T5 series	FLAN-T5-XL	Zero-shot instruction followed by category-specific fine-tuning Synthetic-example augmentation Question-answering style prompting for note-level extraction
Closed-source			
[4,11,64]	GPT series	ChatGPT-4 GPT-4o	Prompt-based inference (multistep, confidence-aware) Integration with retrieval system Clinician-in-the-loop review

a BERT: Bidirectional Encoder Representations from Transformers.

### Model Evaluation

In IE and TC tasks, performance is typically evaluated using a confusion matrix (contingency table) to derive error rates from true positives, false positives, false negatives, and true negatives. Common metrics include sensitivity, recall, specificity, precision (PPV), NPV, and *F*_1_-score. For probabilistic outputs, threshold-independent measures such as AUC and PRAUC are also reported. Most ML studies adopt a hold-out design (training, validation, and test), while cross-validation (CV) estimates predictive error by repeatedly training on subsets and testing on the remainder.

LLM evaluation often follows a multidimensional framework that combines quantitative metrics with expert review. Human assessment—eg, blinded preference tests, distinguishability studies, and Likert-scale rubrics—focuses on clinical utility, coherence, and correction effort. Automated metrics (eg, BERTScore [176], METEOR [177], readability indices [178]) complement human judgment by quantifying semantic fidelity and text quality. Safety and reliability are commonly examined through hallucination and prompt-adherence error analysis, and, when generating synthetic data, statistical comparisons with real-world demographic distributions. Interannotator consistency is frequently measured using Cohen kappa, reflecting variability due to fatigue, interpretation differences, and annotator expertise.

Table 5 summarizes representative model evaluation results across typical IE and TC tasks, including domain-specific evaluation strategies: (1) temporal evaluation, reflecting symptom progression over time (eg, Garriga et al [39], area under the receiver operating characteristic curve=0.865 for mental health crisis prediction); (2) multilabel evaluation, addressing comorbidity (eg, Afshar et al [58], area under the receiver operating characteristic curve=0.88 for alcohol misuse and 0.94 for opioid misuse); (3) severity-aware metrics, particularly relevant for suicide risk where false negatives are high-cost; (4) clinician agreement, benchmarking model outputs against clinician judgments (eg, Leng et al [44], 95% consistency for CI stages classification); (5) cross-domain generalization, comparing performance across conditions or health care systems (eg, Cliffe et al [71]); and (6) unsupervised evaluation, using clinical interpretability of discovered patterns when labels are limited (eg, Andrew et al [46] applying topic modeling and clustering to opioid-related cohorts).

**Table 5.** Results of model evaluation on information extraction and text classification tasks.

Categories and study	Primary aim	Subjects or dataset	Performance or main findings
Rule-based			
[72]	Identify neuropsychiatric symptom domains following COVID-19 hospitalization	6619 patients from 6 Eastern Massachusetts hospitals	The most commonly-documented symptom domains were fatigue (13.4%), mood and anxiety symptoms (11.2%), and impaired cognition (8.0%)
[71]	Characterize each eating disorder patient’s suicidality profile	1126 and 420 patients at WCM^a^ and SLaM^b^	SLaM approach: *F*_1_-score 0.85 versus 0.68; WCM approach: *F*_1_-score 0.87 versus 0.72
Traditional machine learning			
[30]	Predict postdischarge suicides	448,788 VA^c^ patients	AUROC^d^: 0.747‐0.780
[46]	Develop computational phenotypes for patients with opioid-related disorders	82,577 patients from 10 sites within a regional health care network	Reveal 9 distinct opioid-related cohorts
Hybrid			
[57]	Design a data extraction strategy for 21 common physical comorbidities	17,500 individuals at SLaM	Precision rates (*F*_1_-score) above 0.90 for all conditions
[73]	Detect delirium episodes	1,565,678 clinical notes from 10,516 patients from 9 hospitals	Micro *F*_1_-score=0.978; macro *F*_1_-score=0.918
Deep learning			
[39]	Predict mental health crises	59,750 patients from NHS^e^	AUROC: 0.865
[58]	Screen for substance misuse	54,915 and 1991 patients at RUMC and LUMC	AUROCs: 0.88 for alcohol misuse; 0.94 for opioid misuse
Large language models-based			
[65]	Evaluate if AI^f^-generated psychiatric discharge summaries match the quality of those written by residents	20 cases at the Psychiatric University Hospital Zurich	Humans scored significantly higher (3.78 vs 3.12, *P*<.05); Found hallucinations in 40% of AI summaries (37.5% clinically relevant)
[64]	Develop and evaluate a framework to classify CI stages	1002 & 769 patients at MGB	GPT-4o achieved high accuracy (Weighted Kappa 0.95), outperforming BERT and USE models

a WCM: Weill Cornell Medicine.

b SLaM: South London and Maudsley.

c VA: Department of Veterans Affairs.

d AUROC: area under the receiver operating characteristic curve.

e NHS: National Health Service.

f AI: artificial intelligence.

## Discussion

### Principal Results

The application of NLP to psychiatric clinical notes presents unique challenges and opportunities, distinct from other medical domains. This stems from the inherently narrative and subjective nature of mental health documentation, where nuanced descriptions of patients’ experiences and clinicians’ interpretations take precedence over objective measurements. Psychiatric data is often dense, nuanced, and sensitive. Consequently, psychiatric clinical notes demand specialized NLP approaches capable of maintaining context over extensive narratives and extracting meaningful insights from highly subjective content. Parsing these nuanced expressions is crucial for capturing the complexity of a patient’s mental state, which often defies straightforward quantification.

Rule-based methods have long been dominant in psychiatric NLP for IE and TC. Their interpretability, customizability, and efficacy with smaller datasets make them suitable for evolving clinical guidelines. However, a significant drawback of such systems lies in their inability to grasp context, particularly when it comes to negations and expressions of uncertainty. As the complexity of mental health data grows, the field has shifted toward hybrid systems that combine the strengths of rule-based methods and ML approaches. Hybrid systems offer enhanced scalability while retaining transparency and adaptability to clinical needs. Despite this, DL has seen limited adoption in psychiatric clinical notes processing due to challenges such as data scarcity and the demand for model interpretability in clinical decision-making.

In recent years, PLMs based on transformers, such as BERT, have become the cornerstone of psychiatric NLP. By learning linguistic representations directly from large corpora, these models capture semantic nuances far better than previous NLP methods. Their value in mental health applications appears to lie particularly in domain adaptation and task-specific fine-tuning. Domain-adapted variants, including MentalBERT and Bio_ClinicalBERT, extend the utility of general PLMs by continued pretraining on biomedical and clinical corpora, thereby improving the handling of medical terminology, note structure, and psychiatric narrative style. When further fine-tuned for downstream tasks, these models have achieved state-of-the-art performance in tasks such as phenotyping [69], outcome prediction [63], and extracting features [62] from psychiatric clinical notes. However, these advances come with challenges when applied to long longitudinal records, including difficulty handling the exponentially increasing computing requirements in response to the input length.

LLMs offer promising advancements for psychiatric NLP, marking a significant paradigm shift from traditional discriminative tasks to generative and reasoning-based applications. With their flexibility and zero-shot capabilities, LLMs can transform clinical workflows by automating routine tasks, such as generating expert-level discharge summaries [65]. Furthermore, CoT prompting enables the simulation of complex clinical reasoning. Recent studies suggest that LLMs’ use in mental health is expanding beyond summarization to broader forms of clinical assistance [179], including automated coding of clinical encounters, real-time clinical decision support, and the generation of synthetic high-fidelity patient profiles [66]. However, in psychiatric contexts, the risk of hallucination is particularly consequential: models may generate plausible but incorrect interpretations of symptoms, suicidality, psychosis, or treatment adherence, thereby introducing errors into clinical documentation or downstream decision-making. More broadly, the high output variability and limited inherent explainability of LLMs remain major barriers to safe clinical deployment [180]. For this reason, future psychiatric LLM systems will likely require not only retrieval-augmented generation [181], but also stronger human oversight, structured validation, and privacy-preserving deployment strategies [182]. In addition, the increasing reliance on proprietary closed-source models raises important ethical concerns in psychiatry, where clinical notes often contain highly sensitive and stigmatizing information. This consideration has strengthened interest in more transparent and locally deployable alternatives. In particular, parameter-efficient techniques such as LoRA make it possible for smaller fine-tuned models to achieve performance comparable to large proprietary models at a fraction of the cost [67].

Our review reveals a clear evolutionary trajectory in psychiatric NLP methods: from rule-based systems to ML, then to hybrid and DL, and most recently to LLM-based approaches. Each paradigm shift has expanded the capabilities of the field while introducing new trade-offs, as summarized in Table 6. Whereas rule-based methods and traditional ML prioritize interpretability and work well with limited labeled data, DL offers superior contextual understanding at the cost of transparency and data requirements. Hybrid methods help bridge this gap by domain knowledge integration, although they often increase system complexity and maintenance burden. LLMs extend this further by enabling generative applications and few-shot learning, but introduce new concerns around factual reliability and clinical safety. The choice of approach should therefore be guided by the specific clinical task, available resources, data characteristics, and the acceptable trade-off between model performance and interpretability.

**Table 6.** Comparative advantages and disadvantages of natural language processing approaches for psychiatric clinical notes.

NLP^a^ approaches	Advantages	Disadvantages
Rule-based	High interpretability No training data needed Easy to audit Effective with expert curation	Labor-intensive rule creation Poor scalability Brittle to linguistic variation Cannot capture implicit semantics
Traditional ML^b^	Learn patterns from data Rich feature representations Interpretable feature importance Effective on smaller datasets	Requires feature engineering Performance depends on feature quality Limited long-range context modeling
Hybrid	Combine rule interpretability with ML adaptability Balance precision and recall Modular and extensible	Increased system complexity Requires both domain expertise and ML skills Maintenance overhead
Deep learning	Automatic feature learning Capture contextual semantics Domain-adapted BERT^c^ available Strong generalization ability	“Black-box” nature Higher computational cost Performance suffers with limited data Difficulty with long documents
LLMs^d^-based	Zero and few-shot capability Chain-of-thought reasoning High computational efficiency Versatile across tasks Synthetic data generation	Hallucination risk Prompt sensitivity High computational demands Privacy concerns Regulatory uncertainty

a NLP: natural language processing.

b ML: machine learning.

c BERT: Bidirectional Encoder Representations from Transformers.

d LLM: large language model.

The temporal dynamics of mental health conditions further complicate NLP applications in psychiatry. Many psychiatric disorders follow nonlinear progressions, necessitating sophisticated temporal modeling techniques that can track both rapid behavioral changes and long-term symptom trajectories [37,49]. This temporal aspect is vital in psychiatry, where the course of illness can be as informative as the symptoms themselves [183]. Notably, recent NLP research has expanded beyond single-disorder tracking to transdiagnostic approaches. For instance, dynamic temporal network analysis has been successfully applied to model the prodrome of severe mental disorders [51]. By mapping causal pathways among NLP-derived features, researchers identified distinct behavioral communities that consistently precede the onset of full-threshold disorders.

Multimodal integration is another hallmark of psychiatric NLP. Mental health is influenced by a complex interplay of biological, psychological, and social factors, necessitating NLP approaches that can synthesize information from diverse sources, including clinical notes [31,45], genetic studies [184], neuroimaging results [185,186], and even social media interactions [187]. This holistic approach represents a significant departure from traditional, siloed methods of medical data analysis. Integrating NLP-derived clinical features with brain MRI has proven effective in predicting complex conditions like treatment-resistant depression [74]; combining text-based symptom extraction with brain network analysis yields superior predictive performance compared to unimodal methods, validating the additive value of integration.

Public datasets like MIMIC-III are scarce in mental health, meaning most research relies on proprietary institutional data. Research in this domain is distributed across a wide range of institutions, particularly in the US and UK, with key contributors such as major medical centers like SLaM, the VA, and Rush University Medical Center focusing on substance use. However, limited data access is only part of the challenge. Psychiatric clinical notes also reflect important institutional and population-specific differences that may affect model development and generalizability. Clinical notes can vary across institutions in charting style, documentation granularity, terminology, and local diagnostic practice, which may influence the linguistic patterns captured by NLP models. In addition, datasets from individual hospitals or health systems often reflect the demographic, socioeconomic, and clinical characteristics of their local patient populations, introducing potential biases that may reduce the portability of findings across settings. These factors complicate cross-institutional collaboration and create technical barriers to external validation, transfer learning, and domain adaptation. While general health care standards such as OMOP and HL7 FHIR could support improved interoperability, their adoption in psychiatry remains limited, and structural harmonization alone may be insufficient to address variation in narrative documentation. To address these challenges, data-sharing initiatives, privacy-preserving methods such as federated learning, and more systematic cross-site evaluation will be important.

Subjectivity in psychiatric assessments introduces a level of variability that profoundly impacts NLP model development and evaluation. Unlike other medical fields with objective biomarker-based standards, psychiatric NLP must contend with inter-rater variability among clinicians. This challenge has spurred the development of novel evaluation metrics, such as clinician agreement rates and severity-aware measures, particularly crucial in high-stakes scenarios like suicide risk assessment [55].

### Limitations

Although this scoping review was conducted according to the PRISMA-ScR guidelines and a rigorous search strategy, there were some limitations that are worth noting. First, consistent with scoping review methodology, we did not perform a formal risk-of-bias assessment because the aim of this review was to map the scope and methodological landscape of the field rather than to synthesize effect estimates. Second, the requirement for terms specifically related to “psychiatry” or “psychiatric disorder” in the title or abstract means it is possible that some relevant articles focusing on specific conditions without using the broader umbrella terms were not included in this review. Third, the exclusion of non-English text limits the scope of this review to Anglophone clinical settings, potentially overlooking valuable methodological developments in other languages. Finally, this review was conducted in an area of research that is constantly growing and developing and therefore only provides a time-stamped representation of the field.

### Conclusions

Psychiatric NLP is shaped by the distinctive characteristics of mental health documentation: subjective narrative language, longitudinal complexity, and heightened privacy and ethical stakes. The field is progressing from interpretable rule-based systems toward hybrid methods and Transformer-based PLMs, with LLMs enabling new generative and reasoning-based workflows. As these tools evolve, they hold the potential to significantly improve patient care, advance our understanding of mental illness, and ultimately alleviate the burden of those living with mental health conditions. The path forward requires collaborative efforts across health care systems, development of adaptive learning models, and careful ethical implementation. In addressing these challenges, psychiatric NLP not only promises to transform mental health care but also to expand the frontiers of clinical NLP more broadly.

## Supplementary material

10.2196/91249 Multimedia Appendix 1 Search strategy.

10.2196/91249 Multimedia Appendix 2 Data extraction template.

10.2196/91249 Multimedia Appendix 3 Definition of terms.

10.2196/91249 Multimedia Appendix 4 Included records.

10.2196/91249 Multimedia Appendix 5 Distribution of different data sources.

10.2196/91249 Checklist 1 PRISMA-ScR Checklist.

## Abbreviations

ASD: autism spectrum disorder
BERT: CM Encoder Representations from Transformers
COT: chain-of-thought
DL: deep learning
*DSM-V*: *Diagnostic and Statistical Manual of Mental Disorders, Fifth Edition*
EHR: electronic health record
HL7 FHIR: Health Level 7 Fast Healthcare Interoperability Resources
*ICD-9*: *International Classification of Diseases, Ninth Revision*
*ICD:10*: *International Classification of Diseases, Tenth Revision*
IE: information extraction
LDA: latent Dirichlet allocation
LLM: large language model
LoRA: low-rank adaptation
ML: machine learning
NLP: natural language processing
OMOP: observational medical outcomes partnership
PLM: pretrained language model
PRISMA: Preferred Reporting Items for Systematic Reviews and Meta-Analyses
PRISMA-ScR: Preferred Reporting Items for Systematic Reviews and Meta-Analyses Extension for Scoping Reviews
SNOMED-CT: Systematized Nomenclature of Medicine–Clinical Terms
TC: text classification
UMLS: Unified Medical Language System
