# Supplementary material for: Natural Language Processing Applied to Psychiatric Clinical Notes: Scoping Review
Source: JMIR Med Inform. 2026 Jul 10;14:e91249. doi: 10.2196/91249 (PMC13354137; doi:10.2196/91249)
Supplement: Multimedia Appendix 1 [file medinform-v14-e91249-s001.docx]

**Supplemental Files**

Table S2. Search strategy.

| **Source** | **Search terms** | **Records** |
| --- | --- | --- |
| Ovid MEDLINE | ((clinical note* OR exp Electronic Health Records/ OR electronic health record* OR electronic medical record* OR exp Medical Records Systems, Computerized/ OR electronic patient record* OR ATR OR EHR OR EMR OR EPR) AND (exp Natural Language Processing/ OR NLP OR exp Data Mining/) AND (exp Psychiatry/ OR psychiatry disorder.mp.)) AND yr="2021 -Current" | 56 |
| Ovid EMBASE | ('clinic':ab,ti OR 'clinical note*':ab,ti OR 'electronic health record*':ab,ti OR 'electronic medical record*':ab,ti OR 'electronic patient record*':ab,ti OR 'ehr':ab,ti OR 'emr':ab,ti OR 'epr':ab,ti OR 'atr':ab,ti) AND ('nlp':ab,ti OR 'text mining':ab,ti OR 'natural language processing':ab,ti) AND ('psychiatry':ab,ti OR 'psychiatry disorder':ab,ti OR 'psychiatric disorder*':ab,ti) AND [2021-2026]/py | 65 |
| PubMed | (("Electronic Health Records"[Mesh] OR "electronic health record*"[tiab] OR "electronic medical record*"[tiab] OR "electronic patient record*"[tiab] OR "EHR"[tiab] OR "EMR"[tiab] OR "EPR"[tiab] OR "ATR"[tiab] OR "clinical note*"[tiab] OR "clinical text*"[tiab]) AND ("Natural Language Processing"[Mesh] OR "Data Mining"[Mesh] OR "natural language processing"[tiab] OR "NLP"[tiab] OR "text mining"[tiab]) AND ( "Psychiatry"[Mesh] OR "psychiatry"[tiab] OR "psychiatric disorder*"[tiab])) AND (("2021/1/1"[Date - Publication] : "3000"[Date - Publication])) | 96 |
| Scopus | TITLE-ABS-KEY (("clinical note*" OR "electronic health record*" OR "electronic medical record*" OR "electronic patient record*" OR "EHR" OR "EMR" OR "EPR" OR "ATR") AND ("NLP" OR "text mining" OR "natural language processing") AND ("psychiatry" OR "psychiatry disorder" OR "psychiatric disorder")) AND PUBYEAR > 2020 AND PUBYEAR < 2027 | 123 |
| Web of Science | TS = ("clinical note*" OR "electronic health record*" OR "electronic medical record*" OR "electronic patient record*" OR "EHR" OR "EMR" OR "EPR" OR "ATR") AND TS = ("NLP" OR "text mining" OR "natural language processing") AND TS = ("psychiatry" OR "psychiatry disorder" OR "psychiatric disorder") AND PY = (2021-2027) | 70 |
| ACM Digital Library | ("clinical note*" OR "electronic health record*" OR "electronic medical record*" OR "electronic patient record*" OR "EHR" OR "EMR" OR "EPR" OR "ATR") AND ("NLP" OR "text mining" OR "natural language processing") AND ("psychiatry" OR "psychiatry disorder" OR "psychiatric disorder") (Filter: Publication Date: 2021-2026) | 69 |
| Science Direct | ([Title, abstract or author-specified keywords] ("clinical note" OR "electronic health record" OR EHR OR "electronic medical record") AND (NLP OR “text mining” OR "natural language processing") AND (psychiatry OR "psychiatric disorder")) (Filter: Years: 2021, 2026) | 89 |
