# Supplementary material for: Natural Language Processing Applied to Psychiatric Clinical Notes: Scoping Review
Source: JMIR Med Inform. 2026 Jul 10;14:e91249. doi: 10.2196/91249 (PMC13354137; doi:10.2196/91249)
Supplement: Multimedia Appendix 2 [file medinform-v14-e91249-s002.docx]

**Supplemental Files**

Table S3. Data extraction template

| Study Characteristics | Title |  |
| --- | --- | --- |
|  | Author(s) |  |
|  | Lead author contact email |  |
|  | Year |  |
|  | Publication type | journal / conference / workshop |
| Data Characteristics | Data source | database / institution |
|  | Clinical note type |  |
|  | Sample Size |  |
|  |  |  |
| Clinical Context | Psychiatric category |  |
|  | Diagnosis confirmation method |  |
|  | Application domain |  |
| NLP Methodology | NLP task | information extraction / text classification |
|  | NLP method category | Rule-based / Traditional ML / DL / Hybrid / LLM) |
|  | Specific models/tools used |  |
|  | Pre-processing steps |  |
| Ethics and Reproducibility | Ethical considerations |  |
|  | Is the dataset publicly available? |  |
|  | Is the code available for download? |  |
|  |  |  |
| Results | Qualitative description of results |  |
|  | NLP Method Performance indicator (e.g. accuracy/ precision/ recall/ F1 score) |  |
