# Supplementary material for: Natural Language Processing Applied to Psychiatric Clinical Notes: Scoping Review
Source: JMIR Med Inform. 2026 Jul 10;14:e91249. doi: 10.2196/91249 (PMC13354137; doi:10.2196/91249)
Supplement: Multimedia Appendix 3 [file medinform-v14-e91249-s003.docx]

**Supplemental Files**

**Multimedia Appendix 5 - Definition of Terms**

**Machine Learning:** Machine learning is the study and construction of algorithms which can learn from and make predictions from data, and is closely related to computational statistics. Machine learning models can be employed for predictive analysis as well as to reveal hidden insights from trends in data [1].

**Deep Learning:** Deep learning is a branch of machine learning which uses artificial neural networks and machine learning algorithms that have more than one hidden layer [1]. Representative architectures include convolutional neural networks, recurrent neural networks, and Transformers.

**Language Models:** A language model is any computational model that learns the probabilistic structure of natural language — essentially, it estimates how likely a given sequence of words is, or predicts what word is likely to come next. This definition spans both classical statistical approaches (such as n-gram models) and modern neural approaches.

**Pre-trained language models:** A pre-trained language model is a deep neural language model that has been trained on a large, general-purpose text corpus in a self-supervised manner before being adapted to any specific downstream task. The defining characteristic is the two-stage paradigm: the model first acquires broad linguistic knowledge through pre-training on unlabeled text, then transfers this knowledge to specific tasks through fine-tuning or other adaptation methods. Most modern pre-trained language models are built on the Transformer architecture, which can be configured in three principal ways. Encoder-only models (e.g., BERT, RoBERTa) process the entire input bidirectionally and are well suited to language understanding tasks such as classification and information extraction. Decoder-only models (e.g., GPT, LLaMA, Claude) process tokens autoregressively from left to right and are optimized for text generation. Encoder-decoder models (e.g., T5, BART) combine a bidirectional encoder with an autoregressive decoder and are designed for sequence-to-sequence tasks such as translation and summarization.

**Large Language Models:** A large language model is a pre-trained language model scaled to billions of parameters and trained on massive datasets, exhibiting emergent capabilities — such as instruction following, in-context learning, and multi-step reasoning — that smaller pre-trained models do not reliably demonstrate. Contemporary large language models are predominantly based on the decoder-only Transformer architecture. The term "large" refers not merely to parameter count, but to the qualitative capability shift that sufficient scale produces.

[1] P. Ongsulee, "Artificial intelligence, machine learning and deep learning," 2017 15th International Conference on ICT and Knowledge Engineering (ICT&KE), Bangkok, Thailand, 2017, pp. 1-6, doi: 10.1109/ICTKE.2017.8259629.
