# Supplementary material for: Natural Language Processing Applied to Psychiatric Clinical Notes: Scoping Review
Source: JMIR Med Inform. 2026 Jul 10;14:e91249. doi: 10.2196/91249 (PMC13354137; doi:10.2196/91249)
Supplement: Multimedia Appendix 4 [file medinform-v14-e91249-s004.docx]

**Supplemental Files**

Multimedia Appendix 4. Included records (n=101).

(1) Malow BA, Veatch OJ, Niu X, Fitzpatrick KA, Hucks D, Maxwell-Horn A, Davis LK. A practical approach to identifying autistic adults within the electronic health record. Autism Res. 2023;16(1):52-65. [doi: 10.1002/aur.2849] [Medline: 36377765]

(2) Schirle L, Jeffery A, Yaqoob A, Sanchez-Roige S, Samuels DC. Two data-driven approaches to identifying the spectrum of problematic opioid use: a pilot study within a chronic pain cohort. Int J Med Inform. 2021;156:104621. [doi: 10.1016/j.ijmedinf.2021.104621] [Medline: 34673309]

(3) Afshar M, Adelaine S, Resnik F, Mundt MP, Long J, Leaf M, Ampian T, Wills GJ, Schnapp B, Chao M, Brown R, Joyce C, Sharma B, Dligach D, Burnside ES, Mahoney J, Churpek MM, Patterson BW, Liao F. Deployment of real-time natural language processing and deep learning clinical decision support in the electronic health record: pipeline implementation for an opioid misuse screener in hospitalized adults. JMIR Med Inform. 2023;11:e44977. [doi: 10.2196/44977] [Medline: 37079367]

(4) Msosa YJ, Grauslys A, Zhou Y, Wang T, Buchan I, Langan P, Foster S, Walker M, Pearson M, Folarin A, Roberts A, Maskell S, Dobson R, Kullu C, Kehoe D. Trustworthy data and AI environments for clinical prediction: application to crisis-risk in people with depression. IEEE J Biomed Health Inform. 2023;27(11):5588-5598. [doi: 10.1109/JBHI.2023.3312011] [Medline: 37669205]

(5) Joyce C, Markossian TW, Nikolaides J, Ramsey E, Thompson HM, Rojas JC, Sharma B, Dligach D, Oguss MK, Cooper RS, Afshar M. The evaluation of a clinical decision support tool using natural language processing to screen hospitalized adults for unhealthy substance use: protocol for a quasi-experimental design. JMIR Res Protoc. 2022;11(12):e42971. [doi: 10.2196/42971] [Medline: 36534461]

(6) Kessler RC, Bauer MS, Bishop TM, Bossarte RM, Castro VM, Demler OV, Gildea SM, Goulet JL, King AJ, Kennedy CJ, Landes SJ, Liu H, Luedtke A, Mair P, Marx BP, Nock MK, Petukhova MV, Pigeon WR, Sampson NA, Smoller JW, et al. Evaluation of a model to target high-risk psychiatric inpatients for an intensive postdischarge suicide prevention intervention. JAMA Psychiatry. 2023;80(3):230-240. [doi: 10.1001/jamapsychiatry.2022.4634] [Medline: 36652267]

(7) Zhao M, Havrilla J, Peng J, Drye M, Fecher M, Guthrie W, Tunc B, Schultz R, Wang K, Zhou Y. Development of a phenotype ontology for autism spectrum disorder by natural language processing on electronic health records. J Neurodev Disord. 2022;14(1):32. [doi: 10.1186/s11689-022-09442-0] [Medline: 35606697]

(8) Bulik CM, Bertoia ML, Lu M, Seeger JD, Spalding WM. Suicidality risk among adults with binge-eating disorder. Suicide Life Threat Behav. 2021;51(5):897-906. [doi: 10.1111/sltb.12768] [Medline: 34080227]

(9) Chatham AH, Bradley ED, Schirle L, Sanchez-Roige S, Samuels DC, Jeffery AD. Detecting problematic opioid use in the electronic health record: automation of the addiction behaviors checklist in a chronic pain population. medRxiv. Preprint posted online June 12, 2023. [doi: 10.1101/2023.06.08.23290894] [Medline: 37398208]

(10) Shah-Mohammadi F, Cui W, Bachi K, Hurd Y, Finkelstein J. Using natural language processing of clinical notes to predict outcomes of opioid treatment program. In: 2022 44th Annual International Conference of the IEEE Engineering in Medicine & Biology Society (EMBC). 2022:4415-4420. [doi: 10.1109/EMBC48229.2022.9871960] [Medline: 36085745]

(11) Zhu VJ, Lenert LA, Barth KS, Simpson KN, Li H, Kopscik M, Brady KT. Automatically identifying opioid use disorder in non-cancer patients on chronic opioid therapy. Health Informatics J. 2022;28(2):14604582221107808. [doi: 10.1177/14604582221107808] [Medline: 35726687]

(12) Spalding WM, Bertoia ML, Bulik CM, Seeger JD. Treatment characteristics among patients with binge-eating disorder: an electronic health records analysis. Postgrad Med. 2023;135(3):254-264. [doi: 10.1080/00325481.2021.2018255] [Medline: 35037815]

(13) Young M, Holmes NE, Kishore K, Amjad S, Gaca M, Serpa Neto A, Reade MC, Bellomo R. Natural language processing diagnosed behavioural disturbance phenotypes in the intensive care unit: characteristics, prevalence, trajectory, treatment, and outcomes. Crit Care. 2023;27(1):425. [doi: 10.1186/s13054-023-04695-0] [Medline: 37925406]

(14) George A, Johnson D, Carenini G, Eslami A, Ng R, Portales-Casamar E. Applications of aspect-based sentiment analysis on psychiatric clinical notes to study suicide in youth. AMIA Jt Summits Transl Sci Proc. 2021;2021:229-237. [Medline: 34457137]

(15) Garriga R, Buda TS, Guerreiro J, Omaña Iglesias J, Estella Aguerri I, Matić A. Combining clinical notes with structured electronic health records enhances the prediction of mental health crises. Cell Rep Med. 2023;4(11):101260. [doi: 10.1016/j.xcrm.2023.101260] [Medline: 37913776]

(16) Carson NJ, Yang X, Mullin B, Stettenbauer E, Waddington M, Zhang A, Williams P, Rios Perez GE, Cook BL. Predicting adolescent suicidal behavior following inpatient discharge using structured and unstructured data. J Affect Disord. 2024;350:382-387. [doi: 10.1016/j.jad.2023.12.059] [Medline: 38158050]

(17) Levis M, Levy J, Dent KR, Dufort V, Gobbel GT, Watts BV, Shiner B. Leveraging natural language processing to improve electronic health record suicide risk prediction for Veterans Health Administration users. J Clin Psychiatry. 2023;84(4):22m14568. [doi: 10.4088/JCP.22m14568] [Medline: 37341477]

(18) Chen J, Engelhard M, Henao R, Berchuck S, Eichner B, Perrin EM, Sapiro G, Dawson G. Enhancing early autism prediction based on electronic records using clinical narratives. J Biomed Inform. 2023;144:104390. [doi: 10.1016/j.jbi.2023.104390] [Medline: 37182592]

(19) Morrow D, Zamora-Resendiz R, Beckham JC, Kimbrel NA, Oslin DW, Tamang S, Crivelli S. A case for developing domain-specific vocabularies for extracting suicide factors from healthcare notes. J Psychiatr Res. 2022;151:328-338. [doi: 10.1016/j.jpsychires.2022.04.009] [Medline: 35533516]

(20) Levis M, Levy J, Dufort V, Gobbel GT, Watts BV, Shiner B. Leveraging unstructured electronic medical record notes to derive population-specific suicide risk models. Psychiatry Res. 2022;315:114703. [doi: 10.1016/j.psychres.2022.114703] [Medline: 35841702]

(21) D'Souza EW, MacGregor AJ, Markwald RR, Elkins TA, Zouris JM. Investigating insomnia in United States deployed military forces: a topic modeling approach. Sleep Health. 2024;10(1):75-82. [doi: 10.1016/j.sleh.2023.09.014] [Medline: 38071173]

(22) Taylor RA, Gilson A, Schulz W, Lopez K, Young P, Pandya S, Coppi A, Chartash D, Fiellin D, D'Onofrio G. Computational phenotypes for patients with opioid-related disorders presenting to the emergency department. PLoS One. 2023;18(9):e0291572. [doi: 10.1371/journal.pone.0291572] [Medline: 37713393]

(23) Lee DY, Kim N, Park C, Gan SJ, Son SJ, Park RW, Park B. Explainable multimodal prediction of treatment-resistance in patients with depression leveraging brain morphometry and natural language processing. Psychiatry Res. 2024;334:115817. [doi: 10.1016/j.psychres.2024.115817] [Medline: 38430816]

(24) Levis M, Levy J, Dimambro M, Dufort V, Ludmer DJ, Goldberg M, Shiner B. Using natural language processing to evaluate temporal patterns in suicide risk variation among high-risk Veterans. Psychiatry Res. 2024;339:116097. [doi: 10.1016/j.psychres.2024.116097] [Medline: 39083961]

(25) Oh IY, Schindler SE, Ghoshal N, Lai AM, Payne PRO, Gupta A. Extraction of clinical phenotypes for Alzheimer's disease dementia from clinical notes using natural language processing. JAMIA Open. 2023;6(1):ooad014. [doi: 10.1093/jamiaopen/ooad014] [Medline: 36844369]

(26) Arribas M, Oliver D, Patel R, Kornblum D, Shetty H, Damiani S, Krakowski K, Provenzani U, Stahl D, Koutsouleris N, McGuire P, Fusar-Poli P. A transdiagnostic prodrome for severe mental disorders: an electronic health record study. Mol Psychiatry. 2024;29(11):3305-3315. [doi: 10.1038/s41380-024-02533-5] [Medline: 38710907]

(27) Arribas M, Barnby JM, Patel R, McCutcheon RA, Kornblum D, Shetty H, Krakowski K, Stahl D, Koutsouleris N, McGuire P, Fusar-Poli P, Oliver D. Longitudinal evolution of the transdiagnostic prodrome to severe mental disorders: a dynamic temporal network analysis informed by natural language processing and electronic health records. Mol Psychiatry. 2025;30(7):2931-2942. [doi: 10.1038/s41380-025-02896-3] [Medline: 39843546]

(28) Krakowski K, Oliver D, Arribas M, Stahl D, Fusar-Poli P. Dynamic and transdiagnostic risk calculator based on natural language processing for the prediction of psychosis in secondary mental health care: development and internal-external validation cohort study. Biol Psychiatry. 2024;96(7):604-614. [doi: 10.1016/j.biopsych.2024.05.022] [Medline: 38852896]

(29) Hutto A, Zikry TM, Bohac B, Rose T, Staebler J, Slay J, Cheever CR, Kosorok MR, Nash RP. Using a natural language processing toolkit to classify electronic health records by psychiatric diagnosis. Health Informatics J. 2024;30(4):14604582241296411. [doi: 10.1177/14604582241296411] [Medline: 39466373]

(30) Wang T, Codling D, Msosa YJ, Broadbent M, Kornblum D, Polling C, Searle T, Delaney-Pope C, Arroyo B, Maclellan S, Keddie Z, Docherty M, Roberts A, Stewart R, McGuire P, Dobson R, Harland R. VIEWER: an extensible visual analytics framework for enhancing mental healthcare. J Am Med Inform Assoc. 2026;33(1):144-158. [doi: 10.1093/jamia/ocaf010] [Medline: 39847478]

(31) Wang T, Codling D, Bhugra D, Msosa Y, Broadbent M, Patel R, Roberts A, McGuire P, Stewart R, Dobson R, Harland R. Unraveling ethnic disparities in antipsychotic prescribing among patients with psychosis: a retrospective cohort study based on electronic clinical records. Schizophr Res. 2023;260:168-179. [doi: 10.1016/j.schres.2023.08.024] [Medline: 37669576]

(32) Koleck TA, Tatonetti NP, Bakken S, Mitha S, Henderson M, George M, Miaskowski C, Smaldone A, Topaz M. Identifying symptom information in clinical notes using natural language processing. Nurs Res. 2021;70(3):173-183. [doi: 10.1097/NNR.0000000000000488] [Medline: 33196504]

(33) Bendayan R, Kraljevic Z, Shaari S, Das-Munshi J, Leipold L, Chaturvedi J, Mirza L, Aldelemi S, Searle T, Chance N, Mascio A, Skiada N, Wang T, Roberts A, Stewart R, Bean D, Dobson R. Mapping multimorbidity in individuals with schizophrenia and bipolar disorders: evidence from the South London and Maudsley NHS Foundation Trust Biomedical Research Centre (SLAM BRC) case register. BMJ Open. 2022;12(1):e054414. [doi: 10.1136/bmjopen-2021-054414] [Medline: 35074819]

(34) Afshar M, Sharma B, Dligach D, Oguss M, Brown R, Chhabra N, Thompson HM, Markossian T, Joyce C, Churpek MM, Karnik NS. Development and multimodal validation of a substance misuse algorithm for referral to treatment using artificial intelligence (SMART-AI): a retrospective deep learning study. Lancet Digit Health. 2022;4(6):e426-e435. [doi: 10.1016/S2589-7500(22)00041-3] [Medline: 35623797]

(35) Verter V, Fan E, Frank D, Georghiou A. Text mining of outpatient narrative notes to predict the risk of psychiatric hospitalization. Transl Psychiatry. 2025;15(1):60. [doi: 10.1038/s41398-025-03276-9] [Medline: 39979298]

(36) Ford E, Stone J, Oliver D, Fell B, Roque G, Robertson S, Fusar-Poli P, Greenwood K. Local adaptation and validation of a transdiagnostic risk calculator for first episode psychosis using mental health patient records. Front Psychiatry. 2025;16:1584719. [doi: 10.3389/fpsyt.2025.1584719] [Medline: 40766924]

(37) Todorović A, Craig P, Pillinger S, Kontari P, Gibbons S, Bryden L, Franarin T, Uysal C, Roque G, Fell B. Akrivia Health Database—deep patient characterisation using a secondary mental healthcare dataset in England and Wales: cohort profile. BMJ Open. 2024;14(10):e088166. [doi: 10.1136/bmjopen-2024-088166] [Medline: 39419624]

(38) Kulkarni D, Ghosh A, Girdhari A, Liu S, Vance LA, Unruh M, Sarkar J. Enhancing pre-trained contextual embeddings with triplet loss as an effective fine-tuning method for extracting clinical features from electronic health record derived mental health clinical notes. Nat Lang Process J. 2024;6:100045. [doi: 10.1016/j.nlp.2023.100045]

(39) Xie K, Gallagher RS, Shinohara RT, Xie SX, Hill CE, Conrad EC, Davis KA, Roth D, Litt B, Ellis CA. Long-term epilepsy outcome dynamics revealed by natural language processing of clinic notes. Epilepsia. 2023;64(7):1900-1909. [doi: 10.1111/epi.17633] [Medline: 37114472]

(40) Leng Y, He Y, Amini S, Magdamo C, Paschalidis I, Mukerji SS, Moura LMVR, Westover MB, Vranceanu AM, Ritchie CS, Blacker D, Dickson JR, Das S. A GPT-4o-powered framework for identifying cognitive impairment stages in electronic health records. NPJ Digit Med. 2025;8(1):401. [doi: 10.1038/s41746-025-01834-5] [Medline: 40610683]

(41) Schwieger A, Angst K, de Bardeci M, Burrer A, Cathomas F, Ferrea S, Grätz F, Knorr M, Kronenberg G, Spiller T, Troi D, Seifritz E, Weber S, Olbrich S. Large language models can support generation of standardized discharge summaries—a retrospective study utilizing ChatGPT-4 and electronic health records. Int J Med Inform. 2024;192:105654. [doi: 10.1016/j.ijmedinf.2024.105654] [Medline: 39437512]

(42) Warner A, LeDue J, Cao Y, Tham J, Murphy TH. Synthetic patient and interview transcript creator: an essential tool for LLMs in mental health. Front Digit Health. 2025;7:1625444. [doi: 10.3389/fdgth.2025.1625444] [Medline: 41019286]

(43) Gireesh H, Shukla L, Shivaprakash P, Mukherjee A, Chand P, Murthy P. Language models for standardising clinical notes and information extraction in addiction psychiatry—an empirical study. Drug Alcohol Rev. 2026;45(1):e70059. [doi: 10.1111/dar.70059] [Medline: 41158037]

(44) Krishnamoorthy Srinivasan SS, Bahadur A, Singh S, Kedia Gupta S, Jain V, Sinha Deb K, Kumar M, Singh P. Demystifying mental health reports through an LLM-based approach. In: Extended Abstracts of the CHI Conference on Human Factors in Computing Systems (CHI EA '25). 2025. [doi: 10.1145/3706599.3720208]

(45) Hua Y, Blackley S, Shinn A, Skinner J, Moran L, Zhou L. Identifying psychosis episodes in psychiatric admission notes via rule-based methods, machine learning, and pre-trained language models. Res Sq. Preprint posted online March 21, 2024. [doi: 10.21203/rs.3.rs-4126574/v1] [Medline: 38562731]

(46) Patra BG, Lepow LA, Kasi Reddy Jagadeesh Kumar P, Vekaria V, Sharma MM, Adekkanattu P, Fennessy B, Hynes G, Landi I, Sanchez Ruiz JA, Ryu E, Biernacka JM, Nadkarni GN, Talati A, Weissman M, Olfson M, Mann JJ, Zhang Y, Charney AW, Pathak J. Extracting social support and social isolation information from clinical psychiatry notes: comparing a rule-based natural language processing system and a large language model. J Am Med Inform Assoc. 2025;32(1):218-226. [doi: 10.1093/jamia/ocae260] [Medline: 39423850]

(47) Cliffe C, Cusick M, Vellupillai S, Shear M, Downs J, Epstein S, Pathak J, Dutta R. A multisite comparison using electronic health records and natural language processing to identify the association between suicidality and hospital readmission amongst patients with eating disorders. Int J Eat Disord. 2023;56(8):1581-1592. [doi: 10.1002/eat.23980] [Medline: 37194359]

(48) Castro VM, Rosand J, Giacino JT, McCoy TH, Perlis RH. Case-control study of neuropsychiatric symptoms in electronic health records following COVID-19 hospitalization in 2 academic health systems. Mol Psychiatry. 2022;27(9):3898-3903. [doi: 10.1038/s41380-022-01646-z] [Medline: 35705635]

(49) Ge W, Alabsi H, Jain A, Ye E, Sun H, Fernandes M, Magdamo C, Tesh RA, Collens SI, Newhouse A, Moura LMVR, Zafar S, Hsu J, Akeju O, Robbins GK, Mukerji SS, Das S, Westover MB. Identifying patients with delirium based on unstructured clinical notes: observational study. JMIR Form Res. 2022;6(6):e33834. [doi: 10.2196/33834] [Medline: 35749214]

(50) Pozuelo Moyano B, Orgeta V, von Gunten A, Vandel P, Ma R, Stewart R, Mueller C. Treatment-resistant late-life depression prevalence and clinical/sociodemographic correlates: an electronic health records study. J Affect Disord. 2025;381:77-83. [doi: 10.1016/j.jad.2025.03.169] [Medline: 40174785]

(51) Annapragada AV, Donaruma-Kwoh MM, Annapragada AV, Starosolski ZA. A natural language processing and deep learning approach to identify child abuse from pediatric electronic medical records. PLoS One. 2021;16(2):e0247404. [doi: 10.1371/journal.pone.0247404] [Medline: 33630934]

(52) Coon H, Shabalin AA, DiBlasi E, Monson ET, Han S, Kaufman EA, Chen D, Kious B, Molina N, Yu Z, Staley MJ, Crockett DK, Colbert SM, Mullins N, Bakian A, Docherty AR, Keeshin BR. Absence of nonfatal suicidal behavior preceding suicide death reveals differences in clinical risks. Psychiatry Res. 2025;345:116391. [doi: 10.1016/j.psychres.2025.116391] [Medline: 39879864]

(53) Khapre S, Stewart R, Taylor C. An evaluation of symptom domains in the 2 years before pregnancy as predictors of relapse in the perinatal period in women with severe mental illness. Eur Psychiatry. 2021;64(1):e26. [doi: 10.1192/j.eurpsy.2021.18] [Medline: 33775256]

(54) Farran D, Bean D, Wang T, Msosa Y, Casetta C, Dobson R, Teo JT, Scott P, Gaughran F. Anticoagulation for atrial fibrillation in people with serious mental illness in the general hospital setting. J Psychiatr Res. 2022;153:167-173. [doi: 10.1016/j.jpsychires.2022.06.054] [Medline: 35820254]

(55) Mason AJC, Bhavsar V, Botelle R, Chandran D, Li L, Mascio A, Sanyal J, Kadra-Scalzo G, Roberts A, Williams M, Stewart R. Applying neural network algorithms to ascertain reported experiences of violence in routine mental healthcare records and distributions of reports by diagnosis. Front Psychiatry. 2024;15:1181739. [doi: 10.3389/fpsyt.2024.1181739] [Medline: 38445087]

(56) Chatham AH, Bradley ED, Troiani V, Beiler DL, Christy P, Schirle L, Sanchez-Roige S, Samuels DC, Jeffery AD. Automating the addiction behaviors checklist for problematic opioid use identification. JAMA Psychiatry. 2025. [doi: 10.1001/jamapsychiatry.2025.0424] [Medline: 40202749]

(57) Thompson HM, Sharma B, Bhalla S, Boley R, McCluskey C, Dligach D, Churpek MM, Karnik NS, Afshar M. Bias and fairness assessment of a natural language processing opioid misuse classifier: detection and mitigation of electronic health record data disadvantages across racial subgroups. J Am Med Inform Assoc. 2021;28(11):2393-2403. [doi: 10.1093/jamia/ocab148] [Medline: 34383925]

(58) Werbeloff N, Thygesen JH, Hayes JF, Viding EM, Johnson S, Osborn DPJ. Childhood sexual abuse in patients with severe mental illness: demographic, clinical and functional correlates. Acta Psychiatr Scand. 2021;143(6):495-502. [doi: 10.1111/acps.13302] [Medline: 33914899]

(59) Han S, Zhang RF, Shi L, Richie R, Liu H, Tseng A, Quan W, Ryan N, Brent D, Tsui FR. Classifying social determinants of health from unstructured electronic health records using deep learning-based natural language processing. J Biomed Inform. 2022;127:103984. [doi: 10.1016/j.jbi.2021.103984] [Medline: 34951137]

(60) Carrell DS, Cronkite DJ, Shea M, Oliver M, Luce C, Matson TE, Bobb JF, Hsu C, Binswanger IA, Browne KC, Saxon AJ, McCormack J, Jelstrom E, Ghitza UE, Campbell CI, Bradley KA, Lapham GT. Clinical documentation of patient-reported medical cannabis use in primary care: toward scalable extraction using natural language processing methods. Subst Abus. 2022;43(1):917-924. [doi: 10.1080/08897077.2021.1986767] [Medline: 35192769]

(61) Maserejian N, Krzywy H, Eaton S, Galvin JE. Cognitive measures lacking in EHR prior to dementia or Alzheimer's disease diagnosis. Alzheimers Dement. 2021;17(7):1231-1243. [doi: 10.1002/alz.12280] [Medline: 33580915]

(62) Barbour K, Tian N, Yozawitz EG, Wolf S, McGoldrick PE, Sands TT, Nelson A, Basma N, Grinspan ZM. Creating rare epilepsy cohorts using keyword search in electronic health records. Epilepsia. 2023;64(8):2095-2106. [doi: 10.1111/epi.17725] [Medline: 37305974]

(63) Noori A, Magdamo C, Liu X, Tyagi T, Li Z, Kondepudi A, Alabsi H, Rudmann E, Wilcox D, Brenner L, Robbins GK, Moura L, Zafar S, Benson NM, Hsu J, Dickson JR, Serrano-Pozo A, Hyman BT, Blacker D, Westover MB, Mukerji SS, Das S. Development and evaluation of a natural language processing annotation tool to facilitate phenotyping of cognitive status in electronic health records: diagnostic study. J Med Internet Res. 2022;24(8):e40384. [doi: 10.2196/40384] [Medline: 35930338]

(64) Wang B, Miller-Fleming TW, Yu D, Hucks D, Gantz E, Johnston R, Maxwell-Horn A, Cox N, Sutcliffe J, Mathews CA. Development and validation of electronic health record-based ascertainment of obsessive-compulsive disorder cases and controls. medRxiv. Preprint posted online August 5, 2025. [doi: 10.1101/2025.08.05.25332874]

(65) Colbert SMC, Lepow L, Fennessy B, Iwata N, Ikeda M, Saito T, Terao C, Preuss M, Pathak J, Mann JJ. Distinguishing clinical and genetic risk factors for suicidal ideation and behavior in a diverse hospital population. Transl Psychiatry. 2025;15(63). [doi: 10.1038/s41398-025-03287-6]

(66) Hart KL, Pellegrini AM, Forester BP, Berretta S, Murphy SN, Perlis RH, McCoy TH. Distribution of agitation and related symptoms among hospitalized patients using a scalable natural language processing method. Gen Hosp Psychiatry. 2021;68:46-51. [doi: 10.1016/j.genhosppsych.2020.11.003] [Medline: 33310014]

(67) Abramsky S, St Rose S, Heng YW, Vance LA, Zhang L, Chan KM, Wong JG, Kuah S, Low LT, Adamczyk I. Examining differences in clinical and demographic characteristics of patients with post-traumatic stress disorder across adult treatment subgroups based on the NeuroBlu database: a noninterventional, retrospective cohort study. BMJ Open. 2025;15(x):e099711. [doi: 10.1136/bmjopen-2025-099711]

(68) Lin Y, Sharma B, Thompson HM, Boley R, Perticone K, Chhabra N, Afshar M, Karnik NS. External validation of a machine learning classifier to identify unhealthy alcohol use in hospitalized patients. Addiction. 2022;117(4):925-933. [doi: 10.1111/add.15730] [Medline: 34648211]

(69) Afshar M, Sharma B, Bhalla S, Thompson HM, Dligach D, Boley RA, Kishen E, Simmons A, Perticone K, Karnik NS. External validation of an opioid misuse machine learning classifier in hospitalized adult patients. Addict Sci Clin Pract. 2021;16(1):19. [doi: 10.1186/s13722-021-00229-7] [Medline: 33731220]

(70) Wesley EW, Patel I, Kadra-Scalzo G, Pritchard M, Shetty H, Broadbent M, Segev A, Patel R, Downs J, MacCabe JH, Hayes RD, Fonseca de Freitas D. Gender disparities in clozapine prescription in a cohort of treatment-resistant schizophrenia in the South London and Maudsley case register. Schizophr Res. 2021;232:68-76. [doi: 10.1016/j.schres.2021.05.006] [Medline: 34029960]

(71) Zolnoori M, Barron Y, Song J, Noble J, Burgdorf J, Ryvicker M, Topaz M. HomeADScreen: developing Alzheimer's disease and related dementia risk identification model in home healthcare. Int J Med Inform. 2023;177:105146. [doi: 10.1016/j.ijmedinf.2023.105146] [Medline: 37454599]

(72) St Sauver J, Fu S, Sohn S, Weston S, Fan C, Olson J, Thorsteinsdottir B, LeBrasseur N, Pagali S, Rocca W, Liu H. Identification of delirium from real-world electronic health record clinical notes. J Clin Transl Sci. 2023;7(1):e187. [doi: 10.1017/cts.2023.610] [Medline: 37745939]

(73) Stemerman R, Arguello J, Brice J, Krishnamurthy A, Houston M, Kitzmiller R. Identification of social determinants of health using multi-label classification of electronic health record clinical notes. JAMIA Open. 2021;4(3):ooaa069. [doi: 10.1093/jamiaopen/ooaa069] [Medline: 34514349]

(74) Brown AM, White DG, Adams NB, O'Jiaku-Okorie A, Badwe R, Shaikh S, Adegboye A. Identifying co-occurring disorders among patients with an opioid-involved hospital encounter using National Hospital Care Survey data. Vital Health Stat 2. 2022;(199):1-20. [doi: 10.15620/cdc:119155] [Medline: 36043905]

(75) Ariño H, Bae SK, Chaturvedi J, Wang T, Roberts A. Identifying encephalopathy in patients admitted to an intensive care unit: going beyond structured information using natural language processing. Front Digit Health. 2023;5:1085602. [doi: 10.3389/fdgth.2023.1085602] [Medline: 36817845]

(76) Datar S, Lindemann EA, Silverman G, McEwan R, Finzel R, Kotlyar M, Melton GB, Pakhomov SVS. Identifying mentions of life stressors in clinical notes. In: 2021 IEEE 9th International Conference on Healthcare Informatics (ICHI). 2021. [doi: 10.1109/ICHI52183.2021.00033]

(77) Xie F, Ling Grant DS, Chang J, Amundsen BI, Hechter RC. Identifying suicidal ideation and attempt from clinical notes within a large integrated health care system. Perm J. 2022;26(1):85-93. [doi: 10.7812/TPP/21.102] [Medline: 35609158]

(78) Workman TE, Goulet JL, Brandt CA, Warren AR, Eleazer J, Skanderson M, Lindemann L, Blosnich JR, O'Leary J, Zeng-Treitler Q. Identifying suicide documentation in clinical notes through zero-shot learning. Health Sci Rep. 2023;6(9):e1526. [doi: 10.1002/hsr2.1526] [Medline: 37670815]

(79) Shiner B, Levis M, Dufort VM, Patterson OV, Watts BV, DuVall SL, Russ CJ, Maguen S. Improvements to PTSD quality metrics with natural language processing. J Eval Clin Pract. 2022;28(4):520-530. [doi: 10.1111/jep.13587] [Medline: 34303379]

(80) Sheu YH, Magdamo C, Miller M, Smoller JW, Blacker D. Initial antidepressant choice by non-psychiatrists: learning from large-scale electronic health records. Gen Hosp Psychiatry. 2023;81:1-9. [doi: 10.1016/j.genhosppsych.2022.12.004] [Medline: 36652746]

(81) Hanson RF, Zhu V, Are F, Espeleta H, Wallis E, Heider P, Kautz M, Lenert L. Initial development of tools to identify child abuse and neglect in pediatric primary care. BMC Med Inform Decis Mak. 2023;23(1):266. [doi: 10.1186/s12911-023-02361-7] [Medline: 37978368]

(82) Sedgwick R, Bittar A, Kalsi H, Barack T, Downs J, Dutta R. Investigating online activity in UK adolescent mental health patients: a feasibility study using a natural language processing approach for electronic health records. BMJ Open. 2023;13(4):e061640. [doi: 10.1136/bmjopen-2022-061640] [Medline: 37076163]

(83) Burnett A, Chen N, Zeritis S, Ware S, McGillivray L, Shand F, Torok M. Machine learning algorithms to classify self-harm behaviours in New South Wales Ambulance electronic medical records: a retrospective study. Int J Med Inform. 2022;164:104734. [doi: 10.1016/j.ijmedinf.2022.104734] [Medline: 35605390]

(84) Hart KL, Perlis RH, McCoy TH. Mapping of transdiagnostic neuropsychiatric phenotypes across patients in two general hospitals. J Acad Consult Liaison Psychiatry. 2021;62(4):422-431. [doi: 10.1016/j.jaclp.2021.01.002] [Medline: 34219009]

(85) Seker A, Bullock E, Chandler S, Patel R, Quattrone D, Colling C, Sonuga-Barke EJS, Downs J. Mood instability as a transdiagnostic predictor of cannabis use in attention-deficit/hyperactivity disorder and depression: a natural language processing analysis of electronic health records from 13,025 adolescents. Eur Psychiatry. 2025;68(1):e10095. [doi: 10.1192/j.eurpsy.2025.10095] [Medline: 40843511]

(86) Al-Harrasi AM, Iqbal E, Tsamakis K, Lasek J, Gadelrab R, Soysal P, Kohlhoff E, Tsiptsios D, Rizos E, Perera G, Aarsland D, Stewart R, Mueller C. Motor signs in Alzheimer's disease and vascular dementia: detection through natural language processing, co-morbid features and relationship to adverse outcomes. Exp Gerontol. 2021;146:111223. [doi: 10.1016/j.exger.2020.111223] [Medline: 33450346]

(87) Tsui FR, Shi L, Ruiz V, Ryan ND, Biernesser C, Iyengar S, Walsh CG, Brent DA. Natural language processing and machine learning of electronic health records for prediction of first-time suicide attempts. JAMIA Open. 2021;4(1):ooab011. [doi: 10.1093/jamiaopen/ooab011] [Medline: 33758800]

(88) Ridgway JP, Uvin A, Schmitt J, Oliwa T, Almirol E, Devlin S, Schneider J. Natural language processing of clinical notes to identify mental illness and substance use among people living with HIV: retrospective cohort study. JMIR Med Inform. 2021;9(3):e23456. [doi: 10.2196/23456] [Medline: 33739290]

(89) Wang L, Foer D, MacPhaul E, Lo YC, Bates DW, Zhou L. PASCLex: a comprehensive post-acute sequelae of COVID-19 (PASC) symptom lexicon derived from electronic health record clinical notes. J Biomed Inform. 2022;125:103951. [doi: 10.1016/j.jbi.2021.103951] [Medline: 34785382]

(90) Cusick M, Velupillai S, Downs J, Campion TR, Sholle ET, Dutta R, Pathak J. Portability of natural language processing methods to detect suicidality from clinical text in US and UK electronic health records. J Affect Disord Rep. 2022;10:100430. [doi: 10.1016/j.jadr.2022.100430]

(91) Vaci N, Koychev I, Kim CH, Kormilitzin A, Liu Q, Lucas C, Dehghan A, Nenadic G, Nevado-Holgado A. Real-world effectiveness, its predictors and onset of action of cholinesterase inhibitors and memantine in dementia: retrospective health record study. Br J Psychiatry. 2021;218(5):261-267. [doi: 10.1192/bjp.2020.136] [Medline: 32847633]

(92) Meerwijk EL, Tamang SR, Finlay AK, Ilgen MA, Reeves RM, Harris AHS. Suicide theory-guided natural language processing of clinical progress notes to improve prediction of veteran suicide risk: protocol for a mixed-method study. BMJ Open. 2022;12(8):e065088. [doi: 10.1136/bmjopen-2022-065088] [Medline: 35906054]

(93) Chilman N, Song X, Roberts A, Tolani E, Stewart R, Chui Z, Birnie K, Harber-Aschan L, Gazard B, Chandran D, Sanyal J, Hatch S, Kolliakou A, Das-Munshi J. Text mining occupations from the mental health electronic health record: a natural language processing approach using records from the Clinical Record Interactive Search (CRIS) platform in south London, UK. BMJ Open. 2021;11(3):e042274. [doi: 10.1136/bmjopen-2020-042274] [Medline: 33653749]

(94) Costa T, Menzat B, Engelthaler T, Fell B, Franarin T, Roque G, Wei Y, Zhang X, McAllister-Williams RH. The burden associated with, and management of, difficult-to-treat depression in patients under specialist psychiatric care in the United Kingdom. J Psychopharmacol. 2022;36(5):545-556. [doi: 10.1177/02698811221090628] [Medline: 35491923]

(95) Weiner SG, Lo YC, Carroll AD, Zhou L, Ngo A, Hathaway DB, Rodriguez CP, Wakeman SE. The incidence and disparities in use of stigmatizing language in clinical notes for patients with substance use disorder. J Addict Med. 2023;17(4):424-430. [doi: 10.1097/ADM.0000000000001145] [Medline: 37579098]

(96) Panaite V, Devendorf AR, Finch D, Bouayad L, Luther SL, Schultz SK. The value of extracting clinician-recorded affect for advancing clinical research on depression: proof-of-concept study applying natural language processing to electronic health records. JMIR Form Res. 2022;6(7):e34436. [doi: 10.2196/34436] [Medline: 35896020]

(97) Bittar A, Velupillai S, Roberts A, Dutta R. Using general-purpose sentiment lexicons for suicide risk assessment in electronic health records: corpus-based analysis. JMIR Med Inform. 2021;9(4):e22397. [doi: 10.2196/22397] [Medline: 33845694]

(98) Cliffe C, Seyedsalehi A, Vardavoulia K, Bittar A, Velupillai S, Shetty H, Schmidt U, Dutta R. Using natural language processing to extract self-harm and suicidality data from a clinical sample of patients with eating disorders: a retrospective cohort study. BMJ Open. 2021;11(12):e053808. [doi: 10.1136/bmjopen-2021-053808] [Medline: 34880022]

(99) Singleton J, Li C, Akpunonu PD, Abner EL, Kucharska-Newton AM. Using natural language processing to identify opioid use disorder in electronic health record data. Int J Med Inform. 2023;170:104963. [doi: 10.1016/j.ijmedinf.2022.104963] [Medline: 36529028]

(100) Cusick M, Adekkanattu P, Campion TR, Sholle ET, Myers A, Banerjee S, Alexopoulos G, Wang Y, Pathak J. Using weak supervision and deep learning to classify clinical notes for identification of current suicidal ideation. J Psychiatr Res. 2021;136:95-102. [doi: 10.1016/j.jpsychires.2021.01.052] [Medline: 33581461]

(101) Li Z, Kormilitzin A, Fernandes M, Vaci N, Liu Q, Newby D, Goodday S, Smith T, Nevado-Holgado AJ, Winchester L. Validation of UK Biobank data for mental health outcomes: a pilot study using secondary care electronic health records. Int J Med Inform. 2022;160:104704. [doi: 10.1016/j.ijmedinf.2022.104704] [Medline: 35149320]
