# Supplementary material for: Natural Language Processing Applied to Psychiatric Clinical Notes: Scoping Review
Source: JMIR Med Inform. 2026 Jul 10;14:e91249. doi: 10.2196/91249 (PMC13354137; doi:10.2196/91249)
Supplement: Multimedia Appendix 5 [file medinform-v14-e91249-s005.docx]

**Supplemental Files**

**Multimedia Appendix 6 - Distribution of different data sources**


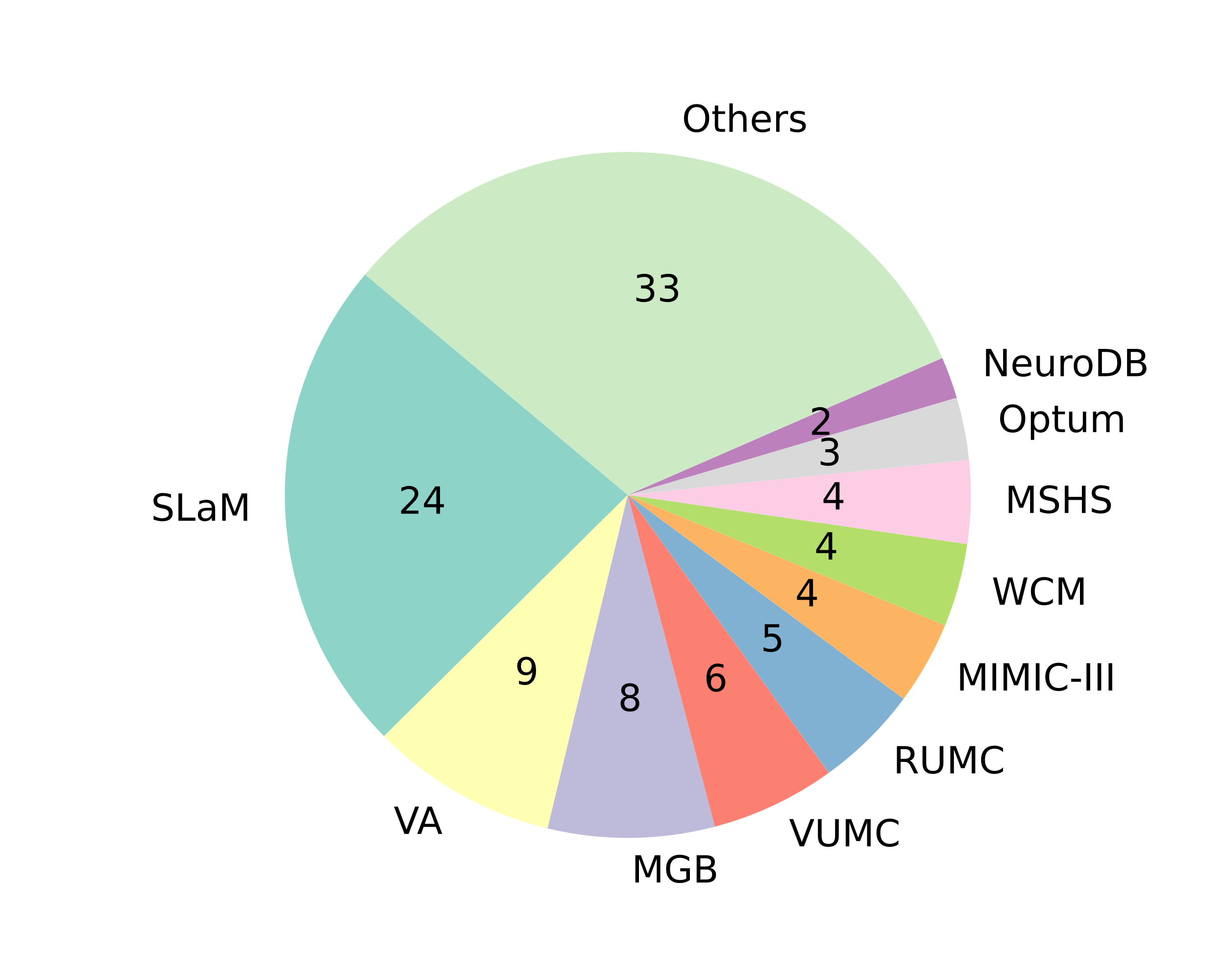


**Figure S1.** Distribution of different data sources. (SLaM: South London and Maudsley NHS Foundation Trust, VA: US Veterans Affairs, MGB: Mass General Brigham health care, VUMC: Vanderbilt University Medical Center, RUMC: Rush University Medical Center, WCM: Weill Cornell Medicine, MSHS: Mount Sinai Health System, NeuroDB: NeuroBlu™ Database, Others: Ajou University School of Medicine, BC Children's Hospital’ s Child and Adolescent Psychiatric Emergency unit, BioMe Biobank, Cambridge Health Alliance, Columbia University Irving Medical Center, Duke University Health System, Kaiser Permanente Southern California, Kaiser Permanente Washington health system, King’s College Hospital, Massachusetts General Hospital, McLean Hospital, Medical University of South Carolina, Mersey Care, MHealth Fairview of the University of Minnesota, Montreal Jewish General Hospital, National Hospital Care Survey, New South Wales Ambulance, New York City Clinical Data Research Network, Psychiatric University Hospital Zurich, Rochester Epidemiology Project, The Children’s Hospital of Philadelphia, The Digital Psychiatry Research Lab in India, The federally mandated Outcome and Assessment Information Set clinical assessment, The UK Biobank, University of Chicago Medicine, University of Kentucky HealthCare, University of North Carolina Health System, University of Pennsylvania, University of Pittsburgh Medical Center, University of Wisconsin Hospital, University of Utah Health Sciences Center, VNS Health, US Department of Defense Military Health System Theater Medical Data Store, Washington University)
